# ﻿Three cryptic *Anaplecta* (Blattodea, Blattoidea, Anaplectidae) species revealed by female genitalia, plus seven new species from China

**DOI:** 10.3897/zookeys.1080.74286

**Published:** 2022-01-04

**Authors:** Jing Zhu, Jiawei Zhang, Xinxing Luo, Zongqing Wang, Yanli Che

**Affiliations:** 1 College of Plant Protection, Southwest University, Beibei, Chongqing 400715, China Southwest University Chongqing China

**Keywords:** ABGD, *Anaplectaomei*, cryptic species, DNA barcodes, female genitalia

## Abstract

Morphological characteristics, including male and female genitalia, combined with DNA barcodes were used to identify 470 *Anaplecta* specimens sampled from China. Ten *Anaplecta* species are new to science, including three cryptic species: *A.paraomei* Zhu & Che, **sp. nov.**, *A.condensa* Zhu & Che, **sp. nov.**, and *A.longihamata* Zhu & Che, **sp. nov.**, which are distinguished mainly by their female genitalia. The other seven new species are as follows: *A.bicruris* Zhu & Che, **sp. nov.**, *A.spinosa* Zhu & Che, **sp. nov.**, *A.ungulata* Zhu & Che, **sp. nov.**, *A.anomala* Zhu & Che, **sp. nov.**, *A.serrata* Zhu & Che, **sp. nov.**, *A.bombycina* Zhu & Che, **sp. nov.**, and *A.truncatula* Zhu & Che, **sp. nov.** This study illustrates that differences in female genitalia can be used to distinguish among species of *Anaplecta*. The female genitalia of 19 Chinese *Anaplecta* species are described and illustrated in this paper.

## ﻿Introduction

The genus *Anaplecta*, has been attributed to the superfamily Blattoidea (Djernæs 2018) based on molecular studies (Djernaes et al. 2015; Wang et al. 2017; Bourguignon et al. 2018). In previous studies, body color and veins were usually used to distinguish *Anaplecta* species (Shelford 1906; Rehn 1916). However, intraspecific variations in costal veins and cross veins of the medio-radia as well as in body color were found, which reduces the value of these characteristics for morphology-based identification (Bruijning, 1948). Almost forty years later, and as for other cockroaches, male genitalia were gradually adopted as the main characters to identify species of *Anaplecta* (Roth 1990, 1996; Lucañas 2016; Deng et al. 2020) .

Deng et al. (2020) established eight *Anaplecta* species from China with the aid of DNA barcodes, which successfully solved several problems in identification. Males and females were difficult to match if only based on morphological characters, and there was intraspecific variation in male genitalia. After careful examination, we found that the samples of *Anaplectaomei* examined in Deng (2020) belong to a complex species containing three species (*A.omei*, *A.paraomei* sp. nov., and *A.condensa* sp. nov.; see below); Deng (2020) had treated the differences in male genitalia as intraspecific variation of *Anaplectaomei*. We re-examined all the samples that had been identified as *Anaplectaomei*, and found some subtle differences in the samples from Libo, Dushan, Mt. Wuliang, and other regions, differences that could either reflect intraspecific variations or interspecific differences.

Therefore, it is necessary to find new morphological characters to identify *Anaplecta* species. Although female genitalia were considered to have fewer useful morphological characters in the taxonomy of cockroaches, Aldrich et al. (2004) successfully identified four *Cryptocercus* species based on female genitalia. Female genitalia have also been used in the identification of *Cryptocercus* (Wang et al. 2015; Bai et al. 2018). Meanwhile, female genitalia of other cockroaches were gradually described in detail and used to distinguish species in Ectobiidae (Bohn et al. 2010; Anisyutkin 2013), Blaberidae (Anisyutkin 2014, 2016), or Blattidae (Grandcolas et al. 2014).

In the present study, we use DNA barcodes combined with morphological characteristics, including male and female genitalia, to comprehensively analyze and identify 470 samples of *Anaplecta*, and to determine whether the samples from Libo, Dushan, Mt. Wuliang should be treated as cryptic species.

## ﻿Materials and methods

### ﻿Morphological study

We examined 470 *Anaplecta* specimens, including 165 females. The measurements are based on examined specimens. The genitalia were soaked in 10% NaOH at 65 °C for 30–35 minutes, then rinsed with distilled water. All segments were dissected and observed in glycerol with a Motic K400 stereomicroscope or a Leica M205A stereomicroscope. Photographs were taken with a Leica M205A stereomicroscope, and edited with Adobe Photoshop CS6. All type materials are deposited at the Institute of Entomology, College of Plant Protection, Southwest University, Chongqing, China (**SWU**).

The terminology for body, male, and female genitalia mainly follows McKittrick (1964), Roth (1990), Wang et al. (2016), and Deng et al. (2020). Terminology for veins follows Li et al. (2018).

### ﻿Abbreviations in this paper are as follows:

**CuA** cubitus anterior;

**CuP** cubitus posterior;

**L1, L2, L3** sclerites of the left phallomere;

**L2d**L2 dorsal;

**L2v**L2 ventral;

**L2vm** median sclerite;

**M** media;

**R1, R2, R3** sclerites of the right phallomere.

### ﻿PCR amplification and sequencing

A total of 38 specimens was used for COI sequencing in this study. Total DNA was extracted from the muscles of the thorax and legs according to the Hipure Tissue DNA MiniKit. Primers for polymerase chain reaction (PCR) were COI-F3 (5’-CAACYAATCATAAAGANATTGGAAC-3’) and COI-R3 (5’-TAAACTTCTGGRTGACCAAARAATCA-3’). The thermal cycling conditions were as follows: initial denaturation 2 min at 98 °C, followed by 35 cycles of 10 s at 98 °C, 10 s, annealing at 49–50 °C, 15 s extension at 72 °C, and a final extension of 2 min at 72 °C; the samples were then held at 8 °C. The PCR products were sequenced by Tsingke (Beijing, China). All sequences were deposited in GenBank with the following accession numbers OL790028-OL790065 (Table 1).

**Table 1.** Samples used in species delimitation.

Species	Location	Voucher number	Accession Number
*A.bicruris* sp. nov.	Mt. Jianfengling, Hainan	SH1(♂)	OL790029
*A.bicruris* sp. nov.	Mt. Jianfengling, Hainan	SH2(♂)	OL790030
*A.bicruris* sp. nov.	Mt. Jianfengling, Hainan	ZJFL4(♀)	OL790036
*A.spinosa* sp. nov.	Mt. Limu, Hainan	N1(♂)	OL790028
*A.spinosa* sp. nov.	Mt. Limu, Hainan	ZLMS2(♀)	OL790038
*A.ungulata* sp. nov.	Xishuangbanna, Yunnan	SP1(♂)	OL790031
*A.ungulata* sp. nov.	Xishuangbanna, Yunnan	ZYRC3(♀)	OL790053
*A.ungulata* sp. nov.	Pu’er, Yunnan	ZMZH1(♂)	OL790048
*A.anomala* sp. nov.	Mt. Wuliang, Yunnan	SP2(♂)	OL790032
*A.anomala* sp. nov.	Mt. Wuliang, Yunnan	ZWLS1(♀)	OL790050
*A.serrata* sp. nov.	Xishuangbanna, Yunnan	SP2_2(♂)	OL790033
*A.serrata* sp. nov.	Xishuangbanna, Yunnan	ZLMC1(♀)	OL790047
*A.serrata* sp. nov.	Naban River, Yunnan	ZGMS1(♂)	OL790046
*A.bombycina* sp. nov.	Pu’er, Yunnan	MZH1(♀)	OL790037
*A.bombycina* sp. nov.	Xishuangbanna, Yunnan	ZSXZ1(♂)	OL790049
*A.bombycina* sp. nov.	Xishuangbanna, Yunnan	SP3(♂)	OL790034
*A.bombycina* sp. nov.	Xishuangbanna, Yunnan	ZYRC2(♀)	OL790052
*A.longihamata* sp. nov.	Mt. Wuliang, Yunnan	SP4(♂)	OL790035
*A.longihamata* sp. nov.	Mt. Wuliang, Yunnan	ZWLS2(♀)	OL790051
*A.paraomei* sp. nov.	Dushan, Guizhou	GZ2(♂)	OL790039
*A.paraomei* sp. nov.	Dushan, Guizhou	DS4_2(♀)	OL790045
*A.paraomei* sp. nov.	Dushan, Guizhou	GZ5(♂)	OL790041
*A.paraomei* sp. nov.	Dushan, Guizhou	GZ6(♀)	OL790042
*A.condensa* sp. nov.	Libo, Guizhou	GZ4(♂)	OL790040
*A.condensa* sp. nov.	Libo, Guizhou	GZ10(♀)	OL790043
*A.condensa* sp. nov.	Guiping, Guangxi	GX8(♂)	OL790044
*A.truncatula* sp. nov.	Chengbu, Hunan	HNSY1(♂)	OL790054
*A.truncatula* sp. nov.	Chengbu, Hunan	HNSY2(♀)	OL790055
*A.omei*	Mt. Jingyun, Chongqing	CQ2(♂)	OL790056
*A.omei*	Mt. Jingyun, Chongqing	CQ5(♀)	OL790057
*A.omei*	Guiping, Guangxi	GX7(♂)	OL790058
*A.omei*	Nanjing, Jiangsu	♂	MT800287
*A.corneola*	Mt. Yinggeling, Hainan	YGL1(♀)	OL790063
*A.corneola*	Mt. Jianfengling, Hainan	♂	MT800293
*A.corneola*	Mount Wuyi, Fujian	♂	MT800296
*A.cruciata*	Mengla, Yunnan	ML3(♀)	OL790061
*A.cruciata*	Mengla, Yunnan	♂	MT800303
*A.cruciata*	Mengla, Yunnan	♂	MT800304
*A.basalis*	Mengla, Yunnan	ML4(♀)	OL790060
*A.basalis*	Xishuangbanna, Yunnan	♂	MT800305
*A.basalis*	Xishuangbanna, Yunnan	♂	MT800309
*A.nigra*	Motuo, Xizang	♂	MT800306
*A.staminiformis*	Mt. Diaoluo, Hainan	DLS3(♀)	OL790062
*A.staminiformis*	Mt. Diaoluo, Hainan	♀	MT800297
*A.staminiformis*	Mt. Limu, Hainan	♂	MT800299
*A.arcuata*	Mt. Limu, Hainan	ZLMS1(♀)	OL790065
*A.arcuata*	Baoting, Hainan	♂	MT800307
*A.arcuata*	Baoting, Hainan	♂	MT800308
*A.strigata*	Pu’er, Yunnan	MZH(♀)	OL790064
*A.strigata*	Mt. Jianfengling, Hainan	♂	MT800291
*A.strigata*	Menglun, Yunnan	♂	MT800292
*A.furcata*	Mt. Dayao, Guangxi	♂	MT800301
*A.furcata*	Mt. Dayao, Guangxi	♂	MT800302
*A.bicolor*	Mengla, Yunnan	ML5(♀)	OL790059
*A.bicolor*	Xishuangbanna, Yunnan	♂	MT800310
*Periplanetaamericana*	Indiana, USA		KC617846
*Periplanetafuliginosa*	Buenos Aires, Argentina		KM577133
*Periplanetaaustralasiae*	China		KF640069

### ﻿Species delimitation and distance analyses

A total of 58 COI sequences was analyzed: 38 sequences of *Anaplecta* species in this study, 17 published sequences of *Anaplecta*, 3 sequences of *Periplaneta* Burmeister, 1838 (as outgroup) downloaded from GenBank (Table 1). All COI sequences were aligned using MEGA 7.0 and adjusted visually after translation into amino acid sequences. Genetic divergence values were quantified based on the Kimura 2-parameter (K2P) distance model (Kimura, 1980). Maximum Likelihood (ML) method was implemented in IQ-TREE (Nguyen et al. 2015) with the GTR+I+G model selected by PartitionFinder v.2.1.1 according to the corrected Akaike Information Criterion (AICc) (Lanfear et al. 2017), and nodal support values were estimated using 1000 bootstrap replicates. We then performed the Automatic Barcode Gap Discovery (ABGD; Puillandre et al. 2012) molecular species delimitation method to provide auxiliary evidence for distinguishing species. As a simple, quick, and efficient method, ABGD is available as a web interface (https://bioinfo.mnhn.fr/abi/public/abgd/abgdweb.html) and was used with default settings, using the Jukes-Cantor (JC69) and p distance model with relative gap width (X = 1.0).

## ﻿Results

### ﻿Morphological delimitation based on external morphology and male genitalia

**Figure 1.**
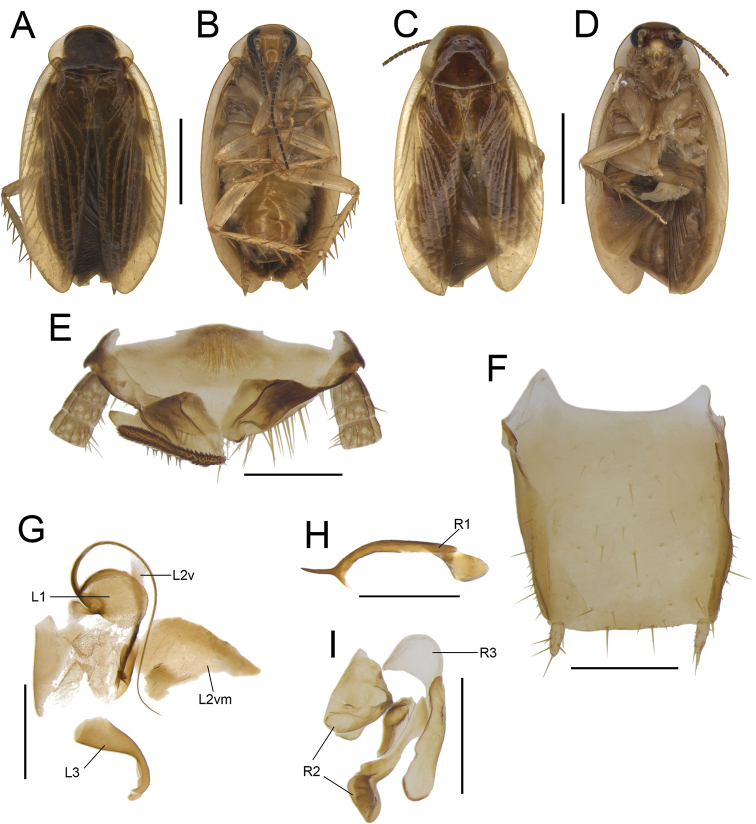
**A, B, E–I***Anaplectaomei* Bey-Bienko, 1958 (CQ2), male SWU-B-B-A060315 **C, D***Anaplectacondensa* Zhu & Che, sp. nov. paratype (GX8), male SWU-B-B-A060126 **A, C** habitus, dorsal view **B, D** habitus, ventral view **E** supra-anal plate, ventral view **F** subgenital plate, dorsal view **G** left phallomere, dorsal view **H, I** right phallomere, ventral view. Scale bars: 2 mm (**A–D**); 0.5 mm (**E–I**). Abbreviations: **L1, L2, L3** sclerites of the left phallomere, **L2v**L2 ventral, **L2vm** median sclerite, **R1, R2, R3** sclerites of the right phallomere.

Observing the external morphological characters and male genitalia of 470 samples of *Anaplecta*, we could easily identify 17 morphospecies. We found there were some differences in the samples from Libo (GZ4), Dushan (GZ2), Mt. Wuliang (SP4), and other regions where samples were initially identified as *Anaplectaomei*. In terms of color, the sample from Libo (GZ4) was grayish brown while those from other regions were mostly yellowish brown (CQ2, GZ2, SP4) (Figs 1A, B, 10A, B, 11A, B, 12A, B). Two samples (CQ2, SP4) have only one paraproct extended backwards, with dense spines on a curly posterior margin, or both paraprocts extended (GZ4), or neither (GZ2) extended. The subgenital plate is sub-rectangular in CQ2 and GZ4 or sub-trapezoidal in SP4 and GZ2. In male genitalia, the L3 has a long uncinate part (SP4) or not (CQ2, GZ2, GZ4), R1 is bifurcated (CQ2, GZ2) or not (SP4, GZ4), R2 consists of three (CQ2, GZ2, SP4) or four (GZ4) sclerites (Figs 1E–I, 10G–K, 11G–K, 12G–K). Due to the instability in body color (Bruijning, 1948) and the intraspecific variations in male genitalia (Deng et al. 2020), it would be premature to use them to distinguish species. Therefore, we have treated them as intraspecific variations of *A.omei*, as in Deng (2020).

### ﻿Phylogenetic analysis based on COI and MOTUs estimations

**Figure 2.**
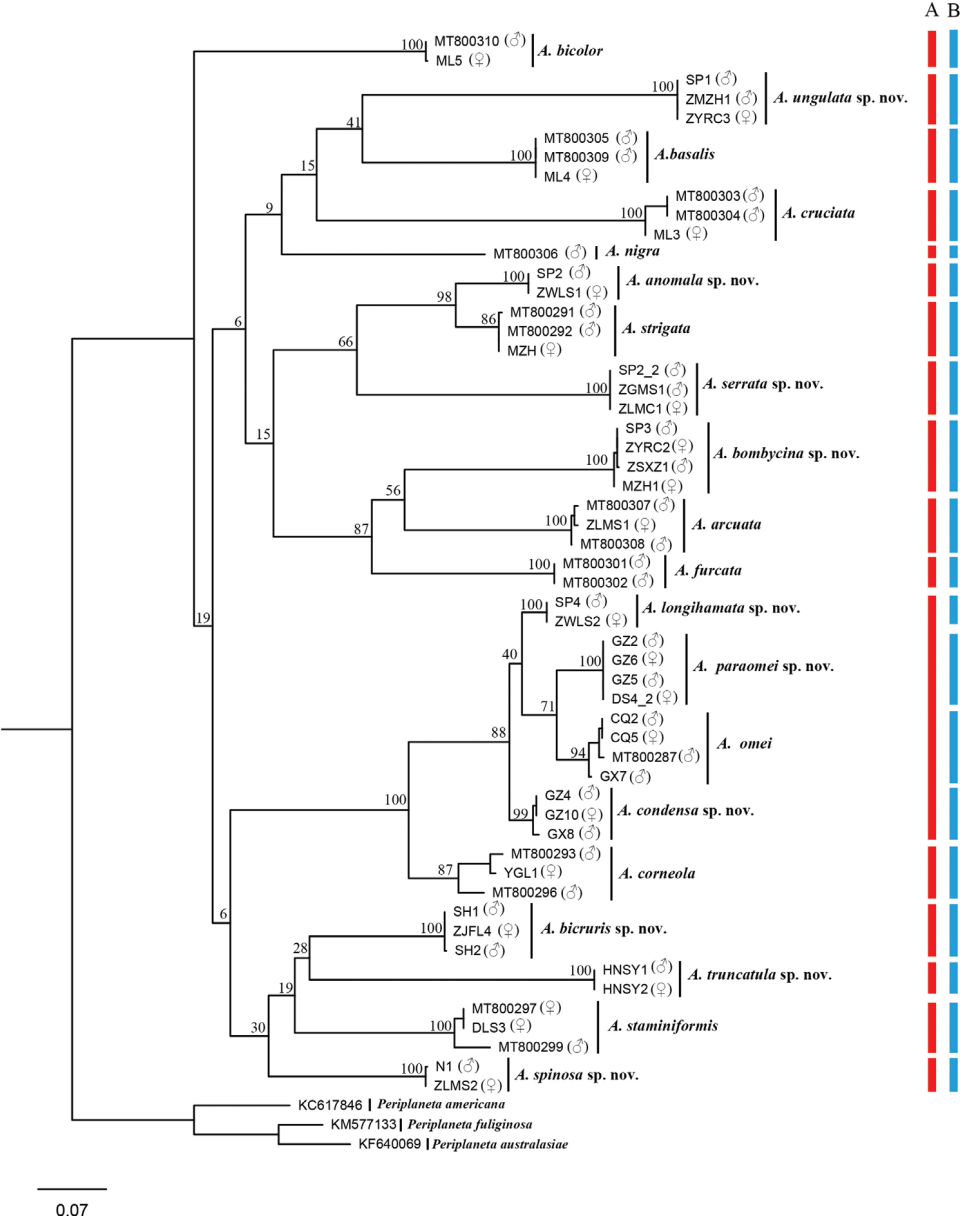
Maximum Likelihood tree derived from COI sequences analyzed with a GTR+I+G model and with 1000 bootstrap replicates. The numbers at nodes are bootstrap values, the sex of the specimens is given in brackets (we checked the voucher specimens of the published sequences to determine whether they were males or females), the red bar indicated morphospecies, the blue bar indicated MOTUs in ABGD (P = 0.0046).

In this study, we acquired 38 COI sequences of *Anaplecta* species. The ML phylogenetic tree showed that males and females of the same morphospecies form monophyletic groups (Fig. 2). Most specific clades have 100 bootstrap values, except *A.strigata* (B = 86), *A.omei* (B = 94), and *A.corneola* (B = 87), indicating that the same morphospecies we identified were well clustered. The relatively low bootstrap values may be caused by the large geographical distances and lack of transitional population. In addition, ABGD analysis produced 20 MOTUs with prior intraspecific divergence (P) = 0.004642, 0.007743, 0.012915, 0.021544, and 0.035938, 17 morphospecies were detected as a single MOTU, but GZ2, GZ5, GZ6, DS4_2, formed one branch, SP4 and ZWLS2 formed a second, and GZ4, GX8, and GZ10 formed a third branch; all were distinct from *A.omei* but more closely related than the other species. The K2P genetic distance between the 38 individuals ranged from 0 to 27.4% (Suppl. material 1: Table S1).

### ﻿Establishment of ten new species based on molecular and morphological data

The results of species delimitation by ABGD were nearly consistent with those by morphological identification (Fig. 2), except 13 samples, which were initially identified as *A.omei* based on external morphological characters and male genitalia, that were divided into 4 MOTUs. But it was insufficient and challenging to distinguish the 13 samples based on the characteristics of male genitalia. Therefore, we examined the females of all *Anaplecta* species from China carefully (except *A.furcata*, *A.malayensis*, *A.simplex*, and *A.arisanica*, for which no female specimen was available), and found there were significant differences among their female genitalia. The sample from Mt. Wuliang (ZWLS2) has a robust and long first valvifer arm, while the first valvifer arm of others (CQ5, GZ10, DS4_2) are short and curled. The sample from Libo (GZ10) has a small and filamentous intercalary sclerite, while the intercalary sclerite of others (ZWLS2, CQ5, DS4_2) are strip-shaped or sheet-like. The anterior arch of the sample from Dushan (DS4_2) has a hip-shaped posterior margin, while that from Mt. Wuliang (ZWLS2) has two transverse finger-like protrusions, and that of CQ5 and GZ10 are smooth. The shape of the basivalvulae are also varied (Fig. 16). Ultimately, we discerned 20 *Anaplecta* species among our 470 samples, including ten new species, using both morphological characteristics and molecular data. The ten new species are *Anaplectabicruris* sp. nov., *A.spinosa* sp. nov., *A.ungulata* sp. nov., *A.anomala* sp. nov., *A.serrata* sp. nov., *A.bombycina* sp. nov., *A.truncatula* sp. nov., *A.longihamata* sp. nov., *A.paraomei* sp. nov., and *A.condensa* sp. nov.

### ﻿Diagnosis of the genus

The characteristics of the external structure and male genitalia are given in Deng et al. (2020) and are therefore not repeated here. Female genitalia: paratergites connected to crosspiece by membrane. First valvifer arm usually short, fused with crosspiece. Anterior margin of anterior arch with weakly sclerotized protrusions, and the shape of basivalvula is always irregular. Spermathecal plate almost merged with basivalvula. Subgenital plate symmetrical. Intersternal fold always simple, sheet-like.

### ﻿Distribution

North America, South America, Africa, Asia, Oceania (Beccaloni, 2014).

### ﻿Key to species of *Anaplecta* in China

**Table:** 

1	Disk of pronotum bicolored	**2**
–	Disk of pronotum unicolored	**6**
2	Disk of pronotum without longitudinal markings	**3**
–	Disk of pronotum with longitudinal markings	**4**
3	Tegmina yellowish brown, 1/3 of the base black (except the lateral margins)	***A.basalis* Bey-Bienko, 1969**
–	Tegmina completely yellowish brown (except the lateral margins)	***A.bicolor* Deng & Che, 2020**
4	Disk of pronotum yellowish brown, with two symmetrical brown markings (Fig. 3C)	***A.bicruris* Zhu & Che, sp. nov.**
–	Disk of pronotum dark brown, with a yellowish brown longitudinal stripe or line on the middle	**5**
5	Tegmina unicolored	***A.strigata* Deng & Che, 2020**
–	Tegmina bicolored, 1/3 of the base darker than remaining parts (except lateral margins and anal field) (Fig. 7E)	***A.anomala* Zhu & Che, sp. nov.**
6	Tegmina with obvious markings	**7**
–	Tegmina without obvious markings	**9**
7	Tegmina yellowish brown, with a nearly oval brown spot at CuP (Fig. 6E)	***A.ungulata* Zhu & Che, sp. nov.**
–	Tegmina yellowish brown, with a subrectangular black spot at base (e.g. Fig. 9E)	**8**
8	R1 needle-shaped (Fig. 9J)	***A.truncatula* Zhu & Che, sp. nov.**
–	R1 arc-shaped	***A.nigra* Deng & Che, 2020**
9	Male paraprocts with dense spines on curly posterior margin (e.g. Figs 1E, 10G)	**10**
–	Male paraprocts not as above	**13**
10	Intercalary sclerite small, nearly filamentous (Fig. 16G, H	***A.condensa* Zhu & Che, sp. nov.**
–	Intercalary sclerite large, strip-shaped or sheet-like	**11**
11	Right first valvifer arm long, lateral edges folded up (Fig. 16A, B)	***A.longihamata* Zhu & Che, sp. nov.**
–	Right first valvifer arm short, lateral edges not folded up	**12**
12	The posterior margin of anterior arch hip-shaped (Fig. 16D, E)	***A.paraomei* Zhu & Che, sp. nov.**
–	The posterior margin of anterior arch smooth (Fig. 16J, K)	***A.omei* Bey-Bienko, 1958**
13	L1 with a long and curved filamentary structure (e.g. Figs 4I, 8I)	**14**
–	L1 with a short and robust uncinate structure	***A.cruciata* Deng & Che, 2020**
14	R1 degraded or merged with L2vm	**15**
–	R1 well developed, not merged with L2vm	**18**
15	Male paraprocts specialized, strip-shaped, with spines on posterior margin (Fig. 4G)	***A.spinosa* Zhu & Che, sp. nov.**
–	Male paraprocts unspecialized	**16**
16	The apex of L2v bifurcated, sheet-like	**17**
–	The apex of L2v not bifurcated, shaped like ‘3’ (Fig. 8I)	***A.bombycina* Zhu & Che, sp. nov.**
17	One sclerite of R2 serrated (Fig. 5J)	***A.serrata* Zhu & Che, sp. nov.**
–	All sclerites of R2 without serration	***A.arcuata* Deng & Che, 2020**
18	R1 curved	**19**
–	R1 straight, cylinder-shaped	***A.staminiformis* Deng & Che, 2020**
19	R1 highly sclerotized horn-shaped	***A.corneola* Deng & Che, 2020**
–	R1 sightly sclerotized arc-shaped	***A.furcata* Deng & Che, 2020**

#### ﻿ Anaplecta bicruris

Taxon classificationAnimaliaBlattodeaAnaplectidae

Zhu & Che sp. nov.

45445876-1B56-5534-9DCE-1BADAF31A745

http://zoobank.org/A05B9533-A9AF-4226-935C-05DF6F6F5693

Figures 3 , 13A–C

##### Type material.

***Holotype***: China • male; Hainan Prov., Ledong County, Mt. Jianfengling; 18°42.63'N, 108°52.75'E; 940–1000 m; 24 June 2020; Yong Li, Jing Zhu leg.; SWU-B-B-A060001.

***Paratypes***: China • 1 male; same data as holotype; SWU-B-B-A060002 • 1 male and 3 females; Hainan Prov., Ledong County, Mt. Jianfengling; 18°42.63'N, 108°52.75'E; 940–960 m; 23 June 2020; Yong Li, Jing Zhu leg.; SWU-B-B-A060003 to 060006 • 5 males; Hainan Prov., Ledong County, Mt. Jianfengling; 18°42.58'N, 108°52.57'E; 940–1000 m; 23 June 2020; Rong Chen, Li-Kang Niu leg.; SWU-B-B-A060007 to 060011.

##### Diagnosis.

This species is similar to *A.corneola* Deng & Che, 2020, but can be distinguished as follows: 1) L2vm stamen-shaped with sharp bifurcation in *A.bicruris* sp. nov., while simple, sheet-like in *A.corneola*; 2) R1 absent in *A.bicruris* sp. nov., while horn-shaped in *A.corneola*; 3) the protrusion of anterior arch horn-shaped in *A.bicruris* sp. nov., while that of *A.corneola* nearly cylindrical; and 4), basivalvula with a backward extension in *A.corneola*, while only curled in *A.bicruris* sp. nov.

##### Etymology.

The specific epithet is derived from the Latin word *bicruris*, meaning that L2vm is bifurcated.

##### Measurements (mm).

Male: pronotum length × width: 1.40–1.49 × 1.84–2.05, tegmina length: 4.97–5.66, overall length: 6.16–6.85. Female: pronotum length × width: 1.34–1.47 × 1.86–2.21, tegmina length: 5.01–5.53, overall length: 6.23–6.75.

##### Description.

**Figure 3.**
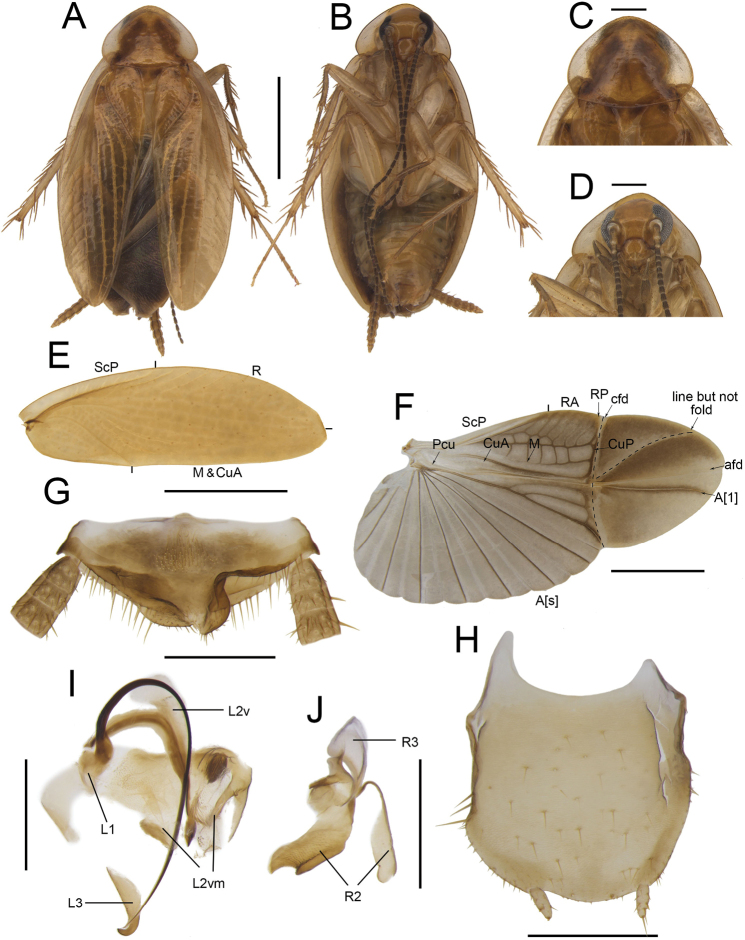
*Anaplectabicruris* Zhu & Che, sp. nov. holotype, male SWU-B-B-A060001 **A** habitus, dorsal view **B** habitus, ventral view **C** pronotum, dorsal view **D** head, ventral view **E** tegmina **F** wings **G** supra-anal plate, ventral view **H** subgenital plate, dorsal view **I** left phallomere, dorsal view **J** right phallomere, ventral view. Scale bars: 2 mm (**A–F**); 0.5 mm (**G–J)**. Abbreviations: **afd** anal fold, **A[1**] the anterior one of the anal vein, **A[s**] the other element of vannal vein, **cfd** cubitus fold, **CuA** cubitus anterior, **CuP** cubitus posterior, **L1, L2, L3** sclerites of the left phallomere, **L2v**L2 ventral, **L2vm** median sclerite, **M** media, **Pcu** postcubitus, **R** radius, **RA** radius anterior, **RP** radius posterior, **R2, R3** sclerites of the right phallomere, **ScP** subcostal posterior.

***Coloration*.** Body light yellowish brown, face yellowish brown (Fig. 3A, B). Antennae brown, maxillary palpus pale brown (Fig. 3D). Pronotum and tegmina light yellowish brown, lateral edges pale or hyaline, pronotum with two symmetrical brown markings (Fig. 3C, E). Hind wings infuscate, costal field and appendicular field darker than remaining parts (Fig. 3F). Abdominal sterna, legs, and cerci yellowish brown (Fig. 3B).

***Head and thorax*.** The distance between antennal sockets slightly narrower than interocular space. Fifth maxillary palpus nearly oval, slightly thicker and wider than others (Fig. 3D). Pronotum nearly sub-elliptical, posterior margin slightly straight (Fig. 3C). Tegmina with slightly indistinct veins; radius posterior veins of hind wings slightly indistinct, without transverse veins between M and CuA (Fig. 3E, F). Front femur Type B_2_ (Fig. 3B). Pulvilli absent, tarsal claws symmetrical.

***Male genitalia*.** Supra-anal plate with sheet-like paraprocts (Fig. 3G). Subgenital plate slightly asymmetrical, the left margin longer than the right and both margins upcurved near the middle; the interstylar margin smooth and curved. Styli medium, length ~ 1/4 of interstylar space (Fig. 3H). L1 small, fan-shaped with a curved and long filamentary structure. L2v slender and curved. L2vm brush-like with a sharp bifurcation. L3 hook-like, stubby with apical part blunt (Fig. 3I). R2 irregular, weakly sclerotized; one of R2 with dense tiny punctuations. R3 slightly curved, sheet-like (Fig. 3J).

***Female genitalia*.** Supra-anal plate nearly symmetrical. Paraprocts broad, not extending to the posterior margin of supra-anal plate. Intercalary sclerite slender, slightly curved. First valve curved. Second valve small, basally fused. Third valve broad. The anterior margin of anterior arch slightly sclerotized, with a horn-shaped protrusion; lateral area with dense tiny punctuation (Fig. 13A, B). Basivalvula irregular, anterior margin curled upward, right lateral deeply concave, lateral area with dense punctuations (Fig. 13A). Spermatheca slightly sclerotized at base. Laterosternal shelf slightly sclerotized, lateral margin slightly curved, with dense spinules at base (Fig. 13C).

##### Distribution.

China (Hainan).

#### ﻿ Anaplecta spinosa

Taxon classificationAnimaliaBlattodeaAnaplectidae

Zhu & Che sp. nov.

F471B62C-7EB2-5F1C-80C9-5DFEB6CA97E0

http://zoobank.org/F0AC2430-A023-4921-AA28-77432A9457B8

Figures 4 , 13D–F

##### Type material.

***Holotype***: China • male; Hainan Prov., Qiongzhong County, Mt. Limu; 19°10.57'N, 109°43.77'E; 650 m; 20 June 2020; Yong Li, Jing Zhu, leg.; SWU-B-B-A060012.

***Paratypes***: China • 1 male and 1 female; same data as holotype; SWU-B-B-A060013 and 060014.

##### Diagnosis.

This species is slightly similar to *A.anncajanoae* Lucañas, 2016, but can be distinguished from the latter by the spines on the left phallomere. It is also similar to *A.cruciata* Deng & Che, 2020 in body color and size, but can be distinguished as follows: 1) sclerites of the left phallomere spinous in *A.spinosa* sp. nov., while spineless in *A.cruciata*; 2) one of R2 with dense punctuations in *A.spinosa* sp. nov., while *A.cruciata* without; 3) anterior margin of anterior arch with a long horn-shaped protrusion in *A.spinosa* sp. nov., that of *A.cruciata* blunter and rounder; and 4) basivalvula nearly triangular in *A.spinosa* sp. nov., while nearly rectangular in *A.cruciata*.

##### Etymology.

The specific epithet is derived from the Latin word *spinosus*, referring to the left phallomere that is spiny.

##### Measurements (mm).

Male: pronotum length × width: 1.19–1.38 × 1.80–1.89, tegmina length: 4.12–4.28, overall length: 5.10–5.57. Female: pronotum length × width: 1.30 × 1.92, tegmina length: 4.29, overall length: 5.55.

##### Description.

**Figure 4.**
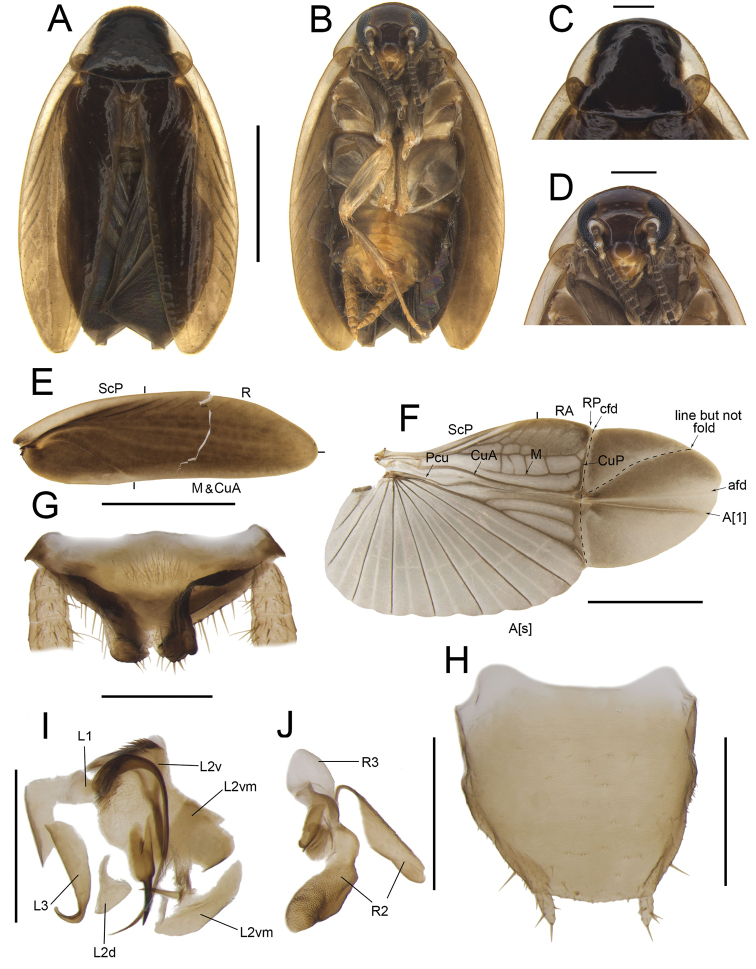
*Anaplectaspinosa* Zhu & Che, sp. nov. holotype, male SWU-B-B-A060012 **A** habitus, dorsal view **B** habitus, ventral view **C** pronotum, dorsal view **D** head, ventral view **E** tegmina **F** wings **G** supra-anal plate, ventral view **H** subgenital plate, dorsal view **I** left phallomere, dorsal view **J** right phallomere, ventral view. Scale bars: 2 mm (**A–F**); 0.5 mm (**G–J)**. Abbreviations: **afd** anal fold, **A[1**] the anterior one of the anal vein, **A[s**] the other element of vannal vein, **cfd** cubitus fold, **CuA** cubitus anterior, **CuP** cubitus posterior, **L1, L2, L3** sclerites of the left phallomere, **L2d**L2 dorsal, **L2v**L2 ventral, **L2vm** median sclerite, **M** media, **Pcu** postcubitus, **R** radius, **RA** radius anterior, **RP** radius posterior, **R2, R3** sclerites of the right phallomere, **ScP** subcostal posterior.

***Coloration*.** Body dark brown, face dark brown, terminal of clypeus and labrum yellowish brown (Fig. 4A, B). Antennae brown, maxillary palpus pale brown (Fig. 4D). Pronotum and tegmina dark brown, lateral edges hyaline (Fig. 4C, E). Hind wings infuscate, costal field and appendicular field darker than remaining parts (Fig. 4F). Center of abdominal sterna yellow, gradually darkening to dark brown to edges. Legs and cerci yellowish brown (Fig. 4B).

***Head and thorax*.** The distance between antennal sockets slightly narrower than interocular space. Fifth maxillary palpus nearly oval, slightly thicker and wider than others (Fig. 4D). Pronotum nearly sub-parabolic, anterior and posterior margins straight (Fig. 4C). Tegmina with slightly indistinct veins; radius posterior veins of hind wings slightly indistinct, with one discontinuous or no transverse veins between M and CuA (Fig. 4E, F). Front femur Type B_2_. Pulvilli absent, tarsal claws symmetrical.

***Male genitalia*.** Supra-anal plate symmetrical. Both paraprocts extend into a strip, with spines on posterior margins (Fig. 4G). Subgenital plate sub-trapezoidal, the center of anterior and interstylar margins straight. Styli medium, length ~ 1/4 of interstylar space (Fig. 4H). L1 fan-shaped, with a curved and long filamentary structure. Terminal of L2v needle-like. L2d small. L2vm with brush-like structure and tapering at terminal. L3 robust, hook-like, apical part enlarged and slightly sharp (Fig. 4I). R2 irregular, weakly sclerotized; one of R2 with dense punctuations. R3 slightly curved, sheet-like (Fig. 4J).

***Female genitalia*.** Supra-anal plate nearly symmetrical. Paraprocts broad, not extending to the posterior margin of supra-anal plate. Intercalary sclerite strip-shaped, slightly curved. First valve robust, with inward protrusions. Second valve small, basally fused. Third valve broad. The anterior margin of anterior arch slightly sclerotized, with a long horn-shaped protrusion, lateral area with dense tiny punctuations (Fig. 13D, E). Basivalvula broad, the right lateral deeply concave, lateral area with dense punctuations (Fig. 13D). Spermatheca slightly sclerotized at base. Laterosternal shelf slightly sclerotized, lateral margin slightly curved, with dense spinules at base (Fig. 13F).

##### Distribution.

China (Hainan).

#### ﻿ Anaplecta serrata

Taxon classificationAnimaliaBlattodeaAnaplectidae

Zhu & Che sp. nov.

F939A451-8DE8-59C5-A763-830F48814B69

http://zoobank.org/5C843FC5-E328-43DB-95F0-61DF51C8B0DD

Figures 5 , 13G–I

##### Type material.

***Holotype***: China • male; Yunnan Prov., Xishuangbanna, Shangyong Town; 21°16.80'N, 101°31.80'E; 870 m; 7 July 2020; Du-Ting Jin, Rong Chen leg.; SWU-B-B-A060015.

***Paratypes***: China • 4 males and 2 females; same data as holotype; SWU-B-B-A060016 to 060021 • 1 male; Yunnan Prov., Jinghong City, Nabanhe Nature Reserve; 22°14.08'N, 100°36.29'E; 1080 m; 3 July 2020; Du-Ting Jin, Yi-Shu Wang, leg.; SWU-B-B-A060022.

##### Diagnosis.

This species is similar to *A.cruciata* Deng & Che, 2020 in body color and size, but can be distinguished as follows: 1) R2 serrated in *A.serrata* sp. nov., while that of *A.cruciata* without serration; 2) anterior margin of anterior arch with a sheet-like protrusion in *A.serrata* sp. nov.; while the protrusions of *A.cruciata* nearly Y-shaped; and 3) basivalvula extremely curled in *A.serrata* sp. nov., while slightly in *A.cruciata*.

##### Etymology.

The specific epithet is derived from the Latin word *serratus*, in reference to the serrated lateral edges of R2.

##### Measurements (mm).

Male: pronotum length × width: 1.12–1.25 × 1.67–1.85, tegmina length: 3.93–4.46, overall length: 5.06–5.53. Female: pronotum length × width: 1.07–1.19 × 1.67–1.69, tegmina length: 4.02–4.06, overall length: 5.00–5.09.

##### Description.

**Figure 5.**
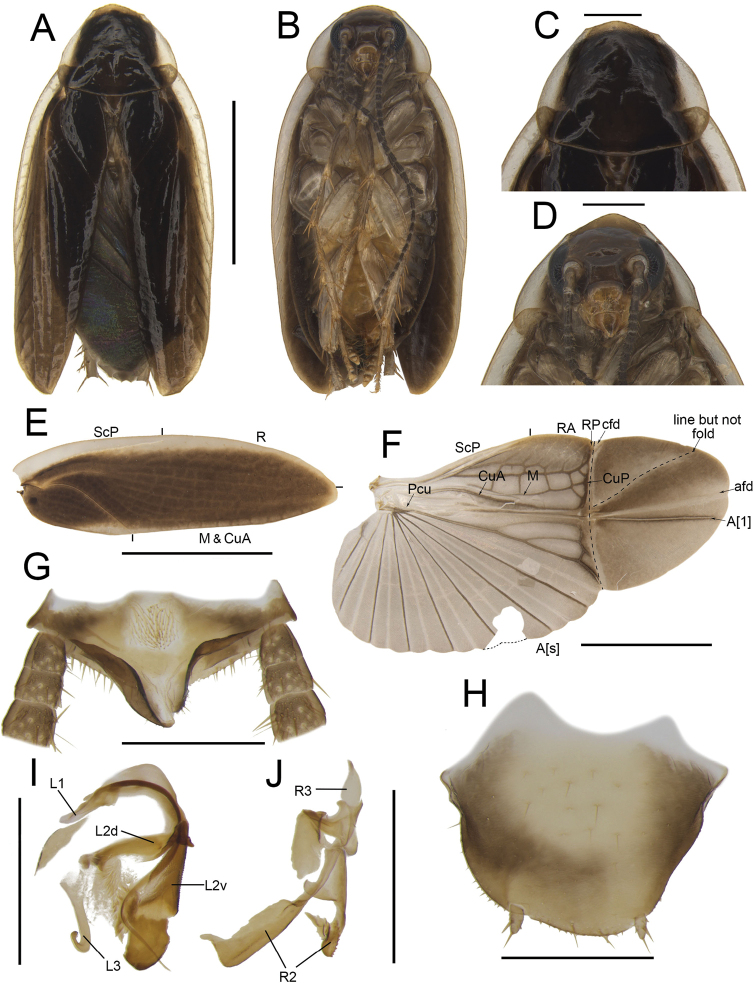
*Anaplectaserrata* Zhu & Che, sp. nov. holotype, male. SWU-B-B-A060015 **A** habitus, dorsal view **B** habitus, ventral view **C** pronotum, dorsal view **D** head, ventral view **E** tegmina **F** wings **G** supra-anal plate, ventral view **H** subgenital plate, dorsal view **I** left phallomere, dorsal view **J** right phallomere, dorsal view. Scale bars: 2 mm (**A–F**); 0.5 mm (**G–J)**. Abbreviations: **afd** anal fold, **A[1**] the anterior one of the anal vein, **A[s**] the other element of vannal vein, **cfd** cubitus fold, **CuA** cubitus anterior, **CuP** cubitus posterior, **L1, L2, L3** sclerites of the left phallomere, **L2d**L2 dorsal, **L2v**L2 ventral, **L2vm** median sclerite, **M** media, **Pcu** postcubitus, **R** radius, **RA** radius anterior, **RP** radius posterior, **R2, R3** sclerites of the right phallomere, **ScP** subcostal posterior.

***Coloration*.** Body dark brown, face dark brown, terminal of clypeus and labrum yellowish brown (Fig. 5A, B). Antennae and maxillary palpus brown (Fig. 5D). Pronotum and tegmina dark brown, lateral edges nearly hyaline (Fig. 5C, E). Hind wings infuscate, costal field and appendicular field darker than remaining parts (Fig. 5F). Center of abdominal sterna yellow, gradually darkening to dark brown to edges. Legs and cerci pale yellowish brown (Fig. 5B).

***Head and thorax*.** The distance between antennal sockets slightly narrower than interocular space. Fifth maxillary palpus nearly triangular, slightly thicker and wider than others (Fig. 5D). Pronotum sub-elliptical, anterior margin straight, posterior margin arcuate (Fig. 5C). Tegmina with slightly indistinct veins, radius posterior veins of hind wings slightly indistinct, with one transverse veins between M and CuA (Fig. 5E,F). Front femur Type B_2_ (Fig. 5B). Pulvilli absent, tarsal claws symmetrical.

***Male genitalia*.** Paraprocts bifurcated at the base: filamentary part short, another part sheet-like (Fig. 5E). Subgenital plate almost symmetrical, anterior margin concave, interstylar margin convex. Styli short, the distance between them long (Fig. 5H). L1 narrow, with a curved and long filamentary structure; L2v broad, folded in the middle. L2d elongated with a sharp horn. L3 small, uncinate part extremely bent (Fig. 5I). R2 irregular, weakly sclerotized; one of R2 with sharp apex, another serrated. R3 slightly curved, sheet-like (Fig. 5J).

***Female genitalia*.** Supra-anal plate nearly symmetrical. Paraprocts broad, not extending to the posterior margin of supra-anal plate. Intercalary sclerite strip-shaped. First valve long. Second valve small, basally fused. Third valve broad. The anterior margin of anterior arch slightly sclerotized, extending forward into a sheet-like protrusion, with wavy depressions. Basivalvula broad, extremely curled, with dense punctuations (Fig. 13G, H). Laterosternal shelf slightly sclerotized, lateral margin slightly curved (Fig. 13I).

##### Distribution.

China (Yunnan).

#### ﻿ Anaplecta ungulata

Taxon classificationAnimaliaBlattodeaAnaplectidae

Zhu & Che sp. nov.

9B4B5462-45B6-54CB-BB2F-58336B37E3C9

http://zoobank.org/9A65A093-36A6-4701-AE54-65F305E8AB2B

Figures 6 , 14A–C

##### Type material.

***Holotype***: China • male; Yunnan Prov., Xishuangbanna, Dadugang Village; 21°59.06'N, 101°64.40'E; 870 m; 14 July 2020; Rong Chen, Li-Kang Niu leg.; SWU-B-B-A060023.

***Paratypes***: China • 10 males and 1 female; same data as holotype; SWU-B-B-A060024 to 060034 • 2 males; Yunnan Prov., Xishuangbanna, Ya’nuo Village; 21°59.70'N, 101°6.02'E; 1212 m; 14 July 2020; Du-Ting Jin, Yi-Shu Wang leg.; SWU-B-B-A060035 and 060036 • 12 males and 5 females; Yunnan Prov., Xishuangbanna, Dadugang Village; 22°16.52'N, 100°55.02'E; 1100 m; 15 July 2020; Rong Chen, Du-Ting Jin leg.; SWU-B-B-A060037 to 060053 • 1 male; Yunnan Prov., Pu’er City, Meizi Lake; 22°44.24'N, 100°58.32'E; 1400 m; 16 July 2020; Du-Ting Jin, Li-Kang Niu, leg.; SWU-B-B-A060054 • 1 male, Yunnan Prov., Pu’er City, Meizi Lake; 22°45.27'N, 100°59.60'E; 1365 m; 17 July 2020; Rong Chen, Yi-Shu Wang, leg.; SWU-B-B-A060055.

##### Diagnosis.

This species can be easily separated from other species by its hoof-shaped right phallomere, and the vestibular sclerite with two serrated and curved long spines.

##### Etymology.

The specific epithet is derived from the Latin word *ungulatus*, referring to the apex of R2 shaped like a pig or horse hoof.

##### Measurements (mm).

Male: pronotum length × width: 1.40–1.47 × 1.95–2.00, tegmina length: 5.31–5.94, overall length: 6.77–7.23. Female: pronotum length × width: 1.21–1.44 × 1.97–2.03, tegmina length: 5.63–5.80, overall length: 6.62–7.11.

##### Description.

**Figure 6.**
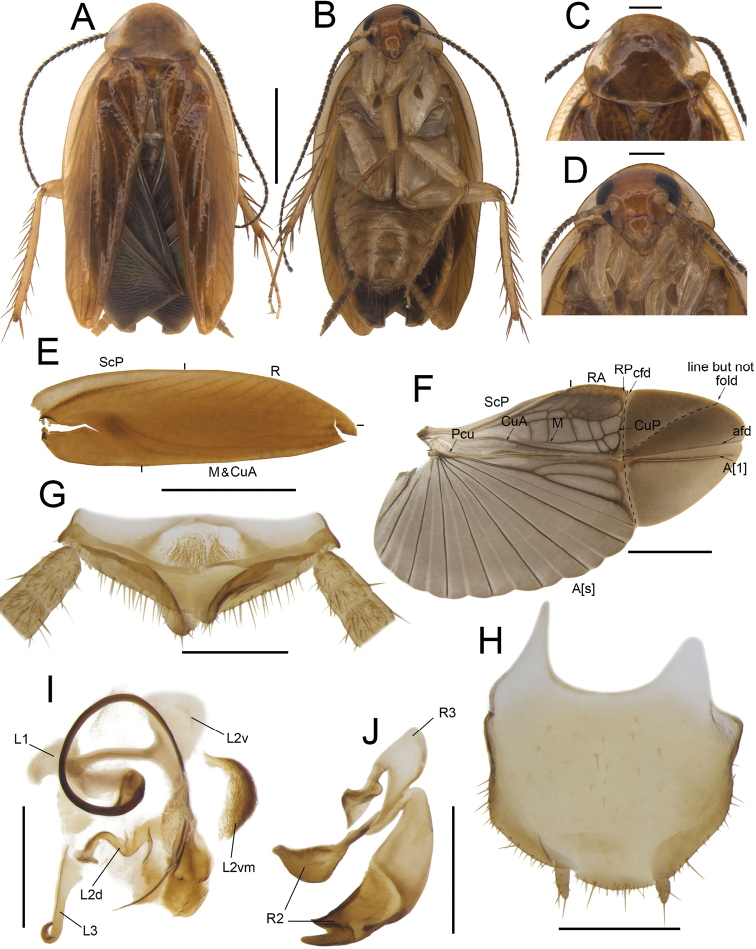
*Anaplectaungulata* Zhu & Che, sp. nov. holotype, male SWU-B-B-A060023 **A** habitus, dorsal view **B** habitus, ventral view **C** pronotum, dorsal view **D** head, ventral view **E** tegmina **F** wings **G** supra-anal plate, ventral view **H** subgenital plate, dorsal view **I** left phallomere, dorsal view **J** right phallomere, dorsal view. Scale bars: 2 mm (**A–F**); 0.5 mm (**G–J)**. Abbreviations: **afd** anal fold, **A[1**] the anterior one of the anal vein, **A[s**] the other element of vannal vein, **cfd** cubitus fold, **CuA** cubitus anterior, **CuP** cubitus posterior, **L1, L2, L3** sclerites of the left phallomere, **L2d**L2 dorsa,l **L2v**L2 ventral, **L2vm** median sclerite, **M** media, **Pcu** postcubitus, **R** radius, **RA** radius anterior, **RP** radius posterior, **R2, R3** sclerites of the right phallomere, **ScP** subcostal posterior.

***Coloration*.** Body yellowish brown, face yellowish brown (Fig. 6A, B). Antennae brown, maxillary palpus pale brown (Fig. 6D). Pronotum and tegmina yellowish brown, lateral edges nearly hyaline, tegmina with a slightly darker marking at the base of mediocubital field (Fig. 6C, E). Hind wings infuscate, costal field and appendicular field darker than remaining parts (Fig. 6F). Abdominal sterna, cerci, and legs yellowish brown (Fig. 6B).

***Head and thorax*.** The distance between antennal sockets slightly narrower than interocular space. Fifth maxillary palpus nearly triangular, slightly thicker and wider than others (Fig. 6D). Pronotum sub-elliptical, anterior margin slightly curved and posterior margin straight (Fig. 6C). Tegmina with slightly indistinct veins; the radius posterior veins of hind wings slightly indistinct, with one or two transverse veins between M and CuA (Fig. 6E, F). Front femur Type B_2_. Pulvilli absent, tarsal claws symmetrical.

***Male genitalia*.** Paraprocts bifurcated at the base: the upper part strip-shaped, approximately the length of paraprocts, the rest sheet-like (Fig. 6G). Subgenital plate asymmetrical, the left margin longer and slender than the right, the interstylar margin curved. The length of styli ~ 1/4 of interstylar space (Fig. 6H). L1 strip-shaped, with extremely curved and long filamentary structure. L2v with a right-angled bifurcation. L2d irregular. L2vm curls and thickens in a crescent shape, with dense spines. L3 slender, apical part extremely bent (Fig. 6I). R2 irregular, weakly sclerotized; one of R2 diverging into two sharp horns at apex. R3 slightly curved, sheet-like (Fig. 6J).

***Female genitalia*.** Supra-anal plate nearly symmetrical. Paraprocts broad, extending to the posterior margin of supra-anal plate. Intercalary sclerite strip-shaped. First valve tubular, with inward protrusions. Second valve small, basally fused. Third valve broad. The anterior margin of anterior arch protrudes in the shape of two triangles. Irregularly shaped basivalvula with dense punctuations, posterior margin curled. The base of vestibular sclerite nearly hyaline, posterior margin bifurcated into two highly sclerotized spines (Fig. 14A, B). Laterosternal shelf nearly hyaline (Fig. 14C).

##### Distribution.

China (Yunnan).

#### ﻿ Anaplecta anomala

Taxon classificationAnimaliaBlattodeaAnaplectidae

Zhu & Che sp. nov.

5E3D0857-73D5-5E52-A592-01E077F29A02

http://zoobank.org/27360C71-7C4F-4174-ADC2-95AC115BE34D

Figures 7 , 14D–F

##### Type material.

***Holotype***: China • male; Yunnan Prov., Pu’er City, Mt. Wuliang; 24°38'N, 100°44'E; 1232 m; 21 July 2020; Li-Kang Niu, Rong Chen, leg.; SWU-B-B-A060056.

***Paratypes***: China • 11 males and 5 females; same data as holotype; SWU-B-B-A060057 to 060072.

##### Diagnosis.

This species is slightly similar to *A.falcifer* Hebard, 1925 but differs in the coloration of pronotum and tegmina. It is also similar to *A.strigata* Deng & Che, 2020 in body color and pronotum, but can be distinguished as follows: 1) the base of the tegmina almost black, while *A.strigata* mostly uniform dark yellowish brown; 2) L2d nearly rectangular in *A.anomala* sp. nov., while slightly bent in *A.strigata*; and 3) anterior margin of anterior arch with a finger-like protrusion, while the protrusion of *A.strigata* nearly wavy.

##### Etymology.

The specific epithet is derived from the Latin word *anomalus*, referring to the left phallomere being different from other species.

##### Measurements (mm).

Male: pronotum length × width: 1.20–1.42 × 1.68–1.95, tegmina length: 4.52–5.49, overall length: 5.94–6.54. Female: pronotum length × width: 1.29 × 1.97, tegmina length: 4.67–5.13, overall length: 5.91–6.22.

##### Description.

**Figure 7.**
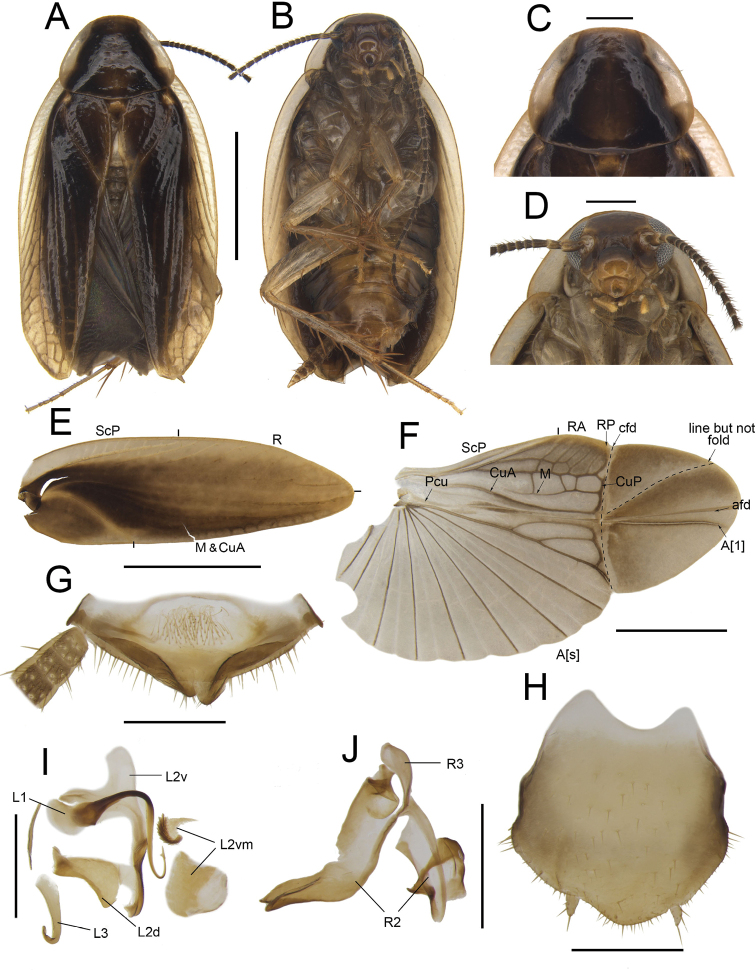
*Anaplectaanomala* Zhu & Che, sp. nov. holotype, male SWU-B-B-A060056 **A** habitus, dorsal view **B** habitus, ventral view **C** pronotum, dorsal view **D** head, ventral view **E** tegmina **F** wings **G** supra-anal plate, ventral view **H** subgenital plate, dorsal view **I** left phallomere, dorsal view **J** right phallomere, dorsal view. Scale bars: 2 mm (**A–F**); 0.5 mm (**G–J)**. Abbreviations: **afd** anal fold, **A[1**] the anterior one of the anal vein, **A[s**] the other element of vannal vein, **cfd** cubitus fold, **CuA** cubitus anterior, **CuP** cubitus posterior, **L1, L2, L3** sclerites of the left phallomere, **L2d**L2 dorsal, **L2v**L2 ventral, **L2vm** median sclerite, **M** media, **Pcu** postcubitus, **R** radius, **RA** radius anterior, **RP** radius posterior, **R2, R3** sclerites of the right phallomere, **ScP** subcostal posterior.

***Coloration*.** Body dark brown, face brown, terminal of clypeus and labrum yellowish brown (Fig. 7A, B). Antennae and maxillary palpus brown (Fig. 7D). Pronotum dark brown, middle part lighter, lateral edges nearly hyaline (Fig. 7C). Tegmina dark brown, lateral edges nearly hyaline, 1/3 of the base darker than remaining parts (except for anal field) (Fig. 7E). Hind wings infuscate, costal field and appendicular field darker than remaining parts (Fig. 7F). Abdominal sterna, legs, and cerci pale yellowish brown (Fig. 7B).

***Head and thorax*.** The distance between antennal sockets slightly narrower than interocular space. Fifth maxillary palpus nearly oval, slightly thicker and wider than others (Fig. 7D). Pronotum sub-elliptical, anterior and posterior margins nearly straight (Fig. 7C). Tegmina with slightly indistinct veins; radius posterior veins of hind wings slightly indistinct, without transverse veins between M and CuA (Fig. 7E, F). Front femur Type B_2_ (Fig. 7B). Pulvilli absent, tarsal claws symmetrical.

***Male genitalia*.** Paraprocts bifurcated at the base: the upper part strip-shaped, length ~ 1/2 of paraprocts, the rest sheet-like (Fig. 7G). Subgenital plate slightly asymmetrical, the left margin slightly wider than the right, the interstylar margin extremely convex. Styli short, the distance between them long (Fig. 7H). L1 fan-shaped, with a curved and long filamentary structure. L2v handle-shaped, with a sharp horn. L2d an approximate rectangle. L2vm with a curled and thickened sclerite, crescent-like with dense spines. L3 medium, hook-like, apical part enlarged and slightly sharp (Fig. 7I). R2 irregular, weakly sclerotized, one of R2 sheet-like, with sharp apex. R3 slightly curved, sheet-like (Fig. 7J).

***Female genitalia*.** Supra-anal plate nearly symmetrical. Paraprocts broad, not extending to the posterior margin of supra-anal plate. Intercalary sclerite slender. First valve tubular. Second valve small, basally fused. Third valve broad. The anterior margin of anterior arch slightly sclerotized, with a finger-like protrusion. Basivalvula broad, nearly triangle, anterior and posterior margin slightly curled (Fig. 14D, E). Vestibular sclerite sheet-like. Laterosternal shelf slightly sclerotized, lateral margin nearly straight (Fig. 14F).

##### Distribution.

China (Yunnan).

#### ﻿ Anaplecta bombycina

Taxon classificationAnimaliaBlattodeaAnaplectidae

Zhu & Che sp. nov.

B94966B8-5D6E-5F2E-BCAB-1AC604B43499

http://zoobank.org/678DC628-4480-4498-9490-9EF66660E8A5

Figures 8 , 14G–I

##### Type material.

***Holotype***: China • male; Yunnan Prov., Xishuangbanna, Dadugang Village; 22°16.52'N, 100°55.02'E; 1100 m; 15 July 2020, Rong Chen, Du-Ting Jin leg.; SWU-B-B-A060073.

***Paratypes***: China • 4 males and 3 females; same data as holotype; SWU-B-B-A060074 and 060080 • 1 female; Yunnan Prov., Pu’er City, Meizi Lake; 22°45.27'N, 100°59.60'E; 1365 m; 17 July 2020; Rong Chen, Yi-Shu Wang, leg.; SWU-B-B-A060081 • 2 female; Yunnan Prov., Xishuangbanna, Ji’nuozu Village; 22°02.44'N, 101°1.81'E; 1100 m; 13 July 2020; Li-Kang Niu, Yi-Shu Wang leg.; SWU-B-B-A060082 and 060083 • 3 males and 1 female; Yunnan Prov., Xishuangbanna, Dadugang Village, 21°59.06'N, 101°64.40'E; 870 m; 14 July 2020; Rong Chen, Li-Kang Niu leg.; SWU-B-B-A060084 to 060087.

##### Diagnosis.

This species can be easily separated from other species by dark brown tegmina and the extremely slender filamentous structure in the male genitalia.

##### Etymology.

The specific epithet is derived from the Latin word *bombycinus*, referring to the slender filamentous structure with which L1 is connected.

##### Measurements (mm).

Male: pronotum length × width: 1.35× 1.57, tegmina length: 4.70, overall length: 6.08. Female: pronotum length × width: 1.42 × 1.68, tegmina length: 4.95, overall length: 6.26.

##### Description.

**Figure 8.**
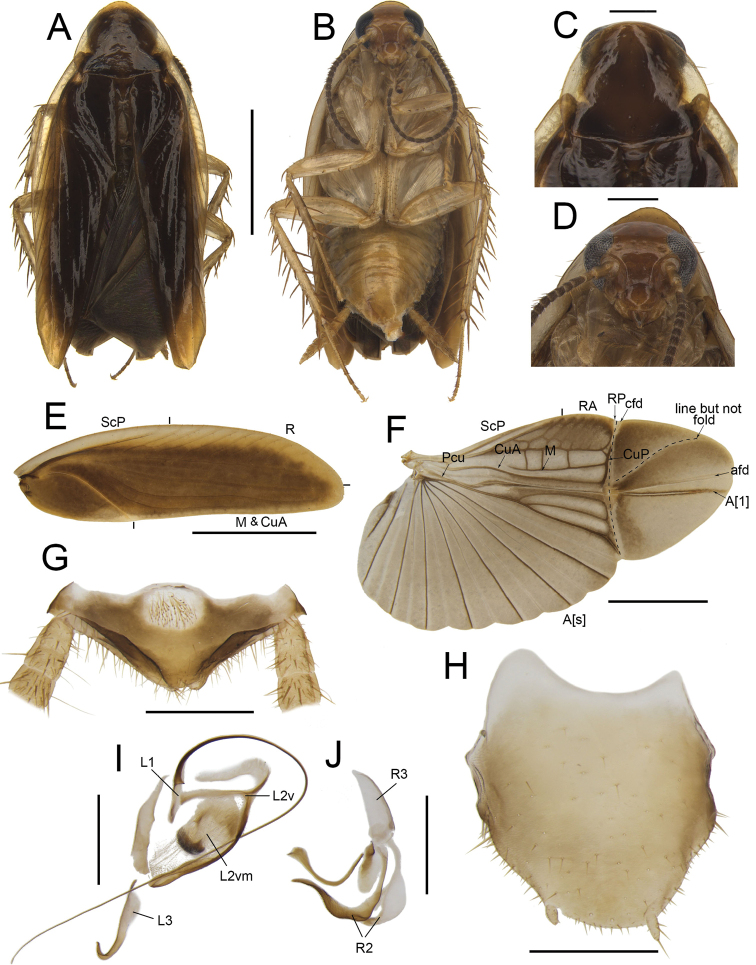
*Anaplectabombycina* Zhu & Che, sp. nov. holotype, male SWU-B-B-A060073 **A** habitus, dorsal view **B** habitus, ventral view **C** pronotum, dorsal view **D** head, ventral view **E** tegmina **F** wings **G** supra-anal plate, ventral view **H** subgenital plate, dorsal view **I** left phallomere, dorsal view **J** right phallomere, dorsal view. Scale bars: 2 mm (**A–F**); 0.5 mm (**G–J)**. Abbreviations: **afd** anal fold, **A[1**] the anterior one of the anal vein, **A[s**] the other element of vannal vein, **cfd** cubitus fold, **CuA** cubitus anterior, **CuP** cubitus posterior, **L1, L2, L3** sclerites of the left phallomere, **L2v**L2 ventral, **L2vm** median sclerite, **M** media, **Pcu** postcubitus, **R** radius, **RA** radius anterior, **RP** radius posterior, **R2, R3** sclerites of the right phallomere, **ScP** subcostal posterior.

***Coloration*.** Body dark brown, face brown (Fig. 8A, B). Antennae and maxillary palpus brown (Fig. 8D). Pronotum and tegmina dark brown, lateral edges hyaline (Fig. 8C, E). Hind wings infuscate, costal field and appendicular field darker than remaining parts (Fig. 8F). Abdominal sterna, legs, and cerci yellowish brown (Fig. 8B).

***Head and thorax*.** The distance between antennal sockets narrower than interocular space. Fifth maxillary palpus nearly triangular, slightly thicker and wider than others (Fig. 8D). Pronotum a semicircle, anterior margin arcuate, posterior margin straight (Fig. 8C). Tegmina with slightly indistinct veins; radius posterior veins of hind wings slightly indistinct, without transverse veins between M and CuA (Fig. 8E, F). Front femur Type B_2_ (Fig. 8B). Pulvilli absent, tarsal claws symmetrical.

***Male genitalia*.** Supra-anal plate with sheet-like paraprocts (Fig. 8G). Subgenital plate asymmetrical, the left margin wider than the right, the interstylar margin convex, skewed to right. The left stylus smaller than the right, the distance between them long (Fig. 8H). L1 small, with a curved and very slender filamentary structure. L2v shaped like ‘3’. L2vm sheet-like, with dense spines. L3 medium, uncinate part with sharp apex (Fig. 8I). R2 irregular, weakly sclerotized. R3 sheet-like (Fig. 8J).

***Female genitalia*.** Supra-anal plate nearly symmetrical. Paraprocts broad, extending to the posterior margin of supra-anal plate. Intercalary sclerite strip-shaped, slightly curved. First valvifer slender. First valve robust. Second valve small, basally fused. Third valve broad. The anterior margin of anterior arch protrudes in the shape of lungs with curved edges (Fig. 14G, H). Basivalvula broad, kidney shaped, posterior margin curled, with spines at left lateral (Fig. 14G). Vestibular sclerite small. Laterosternal shelf slightly sclerotized, lateral margin slightly curved (Fig. 14I).

##### Distribution.

China (Yunnan).

#### ﻿ Anaplecta truncatula

Taxon classificationAnimaliaBlattodeaAnaplectidae

Zhu & Che sp. nov.

9CF1315A-F9AB-59E5-8394-F9FBF3EA458A

http://zoobank.org/B81FCCEA-D820-4488-B570-82F40719F8F9

Figures 9 , 15A–C

##### Type material.

***Holotype***: China • male; Hunan Prov., Shaoyang City, Baimaoping Town; 26°24.90'N, 110°36.04'E; 564 m; 19–21 August 2020; Lu Qiu, leg.; SWU-B-B-A060088.

***Paratypes***: China • 5 males and 3 females; same data as holotype; SWU-B-B-A060089 to 060096.

##### Diagnosis.

This species is similar to *A.japonica* Asahina, 1977 in body color and tegmina marking, but may be distinguished from the latter by the straight interstylar margin, Since *A.japonica* was described by external structures lacking genitalia, a comparison of this part is impossible. It is also similar to *A.nigra* Deng & Che, 2020, but can be distinguished as follows: 1) subgenital plate sub-rectangular in *A.truncatula* sp. nov., while *A.nigra* fan-shaped; 2) R1 needle-shaped in *A.truncatula* sp. nov., while arc-shaped in *A.nigra*; 3) anterior margin of anterior arch with a strip-shaped protrusion in *A.truncatula* sp. nov., while the protrusion of *A.nigra* triangular; and 4) vestibular sclerite with two long spines in *A.nigra*, *A.truncatula* sp. nov. without.

##### Etymology.

The specific epithet is derived from the Latin word *truncatulus*, referring to the truncated end of the bifurcation of the paraprocts.

##### Measurements (mm).

Male: pronotum length × width: 1.28–1.37 × 1.98–2.05, tegmina length: 5.21–5.24, overall length: 6.23–6.32. Female: pronotum length × width: 1.37–1.48 × 1.97–2.13, tegmina length: 5.37–5.46, overall length: 6.58–6.70.

##### Description.

**Figure 9.**
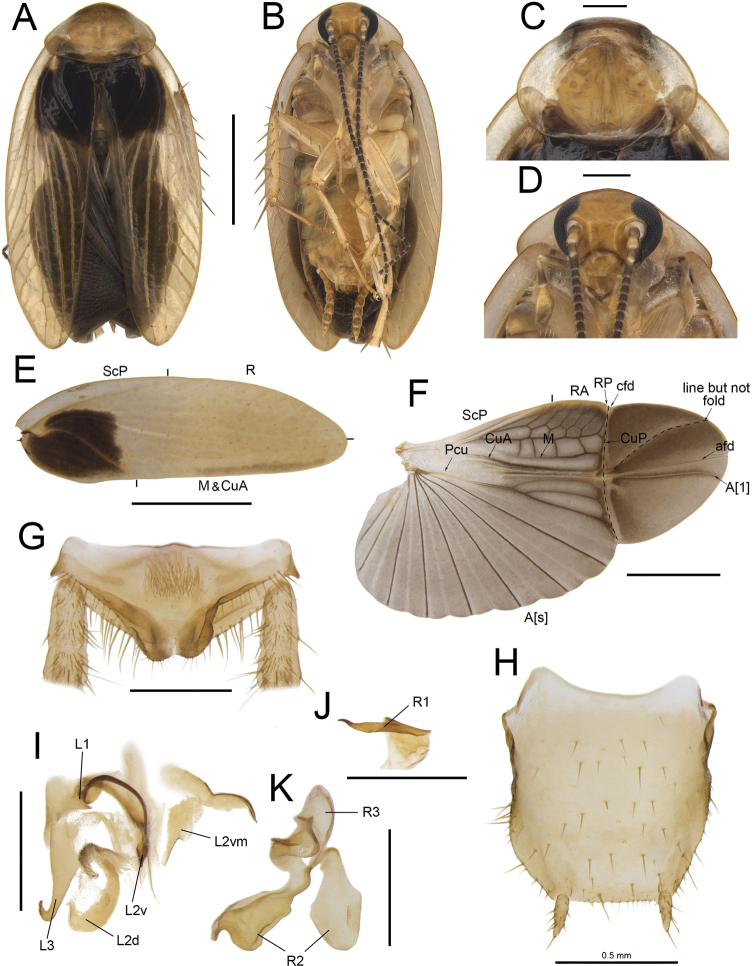
*Anaplectatruncatula* Zhu & Che, sp. nov. holotype, male SWU-B-B-A060088 **A** habitus, dorsal view **B** habitus, ventral view **C** pronotum, dorsal view **D** head, ventral view **E** tegmina **F** wings **G** supra-anal plate, ventral view **H** subgenital plate, dorsal view **I** left phallomere, dorsal view **J–K** right phallomere, dorsal view. Scale bars: 2 mm (**A–F**); 0.5 mm (**G–K)**. Abbreviations: **afd** anal fold, **A[1**] the anterior one of the anal vein, **A[s**] the other element of vannal vein, **cfd** cubitus fold, **CuA** cubitus anterior, **CuP** cubitus posterior, **L1, L2, L3** sclerites of the left phallomere, **L2d**L2 dorsal, **L2v**L2 ventral, **L2vm** median sclerite, **M** media, **Pcu** postcubitus, **R** radius, **RA** radius anterior, **RP** radius posterior, **R1, R2, R3** sclerites of the right phallomere, **ScP** subcostal posterior.

***Coloration*.** Body pale yellowish brown, face yellow (Fig. 9A, B). Antennae and maxillary palpus brown (Fig. 9D). Pronotum yellowish brown, lateral edges hyaline (Fig. 9C). Tegmina light yellowish brown, lateral edges pale or hyaline, 1/3 of the base black (Fig. 9E). Hind wings infuscate, costal field and appendicular field darker than remaining parts (Fig. 9F). Abdominal sterna, legs, and cerci yellow (Fig. 9B).

***Head and thorax*.** The distance between antennal sockets narrower than interocular space. Fifth maxillary palpus nearly triangular, slightly thicker and wider than others (Fig. 9D). Pronotum subelliptic, posterior margin straight, lateral margin protruding and arc-shaped (Fig. 9C). Tegmina with indistinct veins, the radius posterior veins of hind wings distinct, no transverse veins between M and CuA (Fig. 9E, F). Front femur Type B_2_ (Fig. 9B). Pulvilli absent, tarsal claws symmetrical.

***Male genitalia*.** Paraprocts bifurcated at the base: the strip-shaped part truncated, the rest sheet-like (Fig. 9G). Subgenital plate sub-rectangular, the center of anterior slightly concave, interstylar margin straight. Styli long, length ~ 1/2 of interstylar space (Fig. 9H). L1 small, with curved and long filamentary structure. L2v bifurcated, with sharp apex. L2d narrow, nearly meniscus-shaped. L2vm sheet-like, irregular. L3 robust, uncinate part slightly sharp (Fig. 9I). R1 needle-shaped, the proximal part sharply tapered and highly sclerotized (Fig. 9J). R2 irregular, weakly sclerotized. R3 slightly curved, sheet-like (Fig. 9K).

***Female genitalia*.** Supra-anal plate nearly symmetrical. Paraprocts broad, not extending to the posterior margin of supra-anal plate. Intercalary sclerite short, nearly spindle-shaped. Right first valvifer finger-like. First valve robust. Second valve small, basally fused. Third valve broad. The anterior margin of anterior arch slightly sclerotized, with a bifurcated strip-shaped protrusion (Fig. 15A, B). Basivalvula irregular, posterior margin and center with dense punctuations, the left of anterior margin extending back, connecting to crosspiece by membrane (Fig. 15A). Laterosternal shelf slightly sclerotized, lateral margin slightly curved, with dense spinules at lateral base (Fig. 15C).

##### Distribution.

China (Hunan).

#### ﻿ Anaplecta longihamata

Taxon classificationAnimaliaBlattodeaAnaplectidae

Zhu & Che sp. nov.

74BEBACE-956E-54F1-9E66-95A5706DA115

http://zoobank.org/648EBFA2-6972-4528-8C00-886A256949C3

Figures 10 , 16A–C

##### Type material.

***Holotype***: China • male; Yunnan Prov., Pu’er City, Mt. Wuliang; 24°38'N, 100°44'E; 1232 m, 21 July 2020; Li-Kang Niu, Rong Chen leg.; SWU-B-B-A06097.

***Paratypes***: China • 1 male and 1 female; same data as holotype; SWU-B-B-A06098 and 06099 • 2 males; Yunnan Prov., Xishuangbanna, Dadugang Village; 21°59.06'N, 101°64.40'E; 870 m; 14 July 2020; Rong Chen, Li-Kang Niu leg.; SWU-B-B-A06100 and 060101 • 2 males; Yunnan Prov., Xishuangbanna, Dadugang Village; 22°16.52'N, 100°55.02'E; 15 July 2020; Rong Chen, Du-Ting Jin leg.; SWU-B-B-A060102 and 060103.

##### Measurements (mm).

Male: pronotum length × width: 1.39–1.53 × 1.94–2.03, tegmina length: 5.17–5.76, overall length: 6.57–7.09. Female: pronotum length × width: 1.42 × 1.92, tegmina length: 5.12, overall length: 6.43.

##### Diagnosis.

This species is similar to *A.omei* Bey-Bienko, 1958, but can be distinguished as follows: 1) right paraproct long hooked in *A.longihamata* sp. nov., while sheet-like in *A.omei*; 2) R1 bifurcated in *A.omei*, while unbranched in *A.longihamata* sp. nov.;3) anterior arch with two transversely finger-like protrusions in *A.longihamata* sp. nov., while *A.omei* without; and 4) first valvifer arm lateral edges folded up in *A.longihamata* sp. nov., while not folded in *A.omei* .

##### Etymology.

The specific epithet is derived from the Latin words *longi* and *hamatus*, referring to the right paraproct extended backward in a long hook shape.

##### Description.

**Figure 10.**
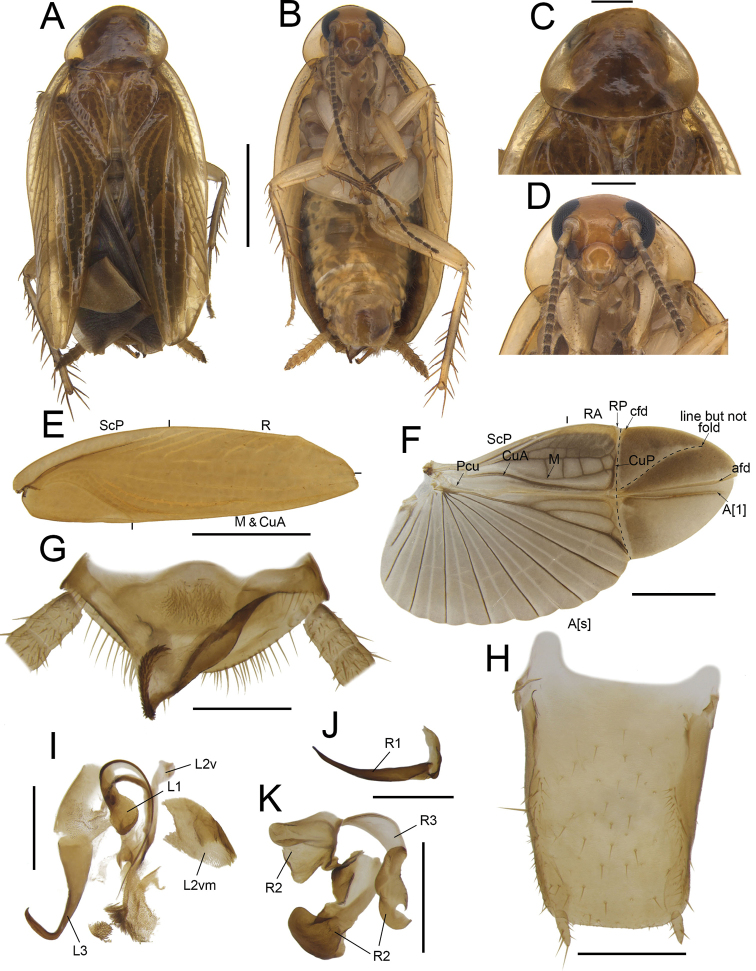
*Anaplectalongihamata* Zhu & Che, sp. nov. holotype (SP4), male SWU-B-B-A06097 **A** habitus, dorsal view **B** habitus, ventral view **C** pronotum, dorsal view **D** head, ventral view **E** tegmina **F** wings **G** supra-anal plate, ventral view **H** subgenital plate, dorsal view **I** left phallomere, dorsal view **J, K** right phallomere **J** dorsal view **K** ventral view. Scale bars: 2 mm (**A–F**); 0.5 mm (**G–K)**. Abbreviations: **afd** anal fold, **A[1**] the anterior one of the anal vein, **A[s**] the other element of vannal vein, **cfd** cubitus fold, **CuA** cubitus anterior, **CuP** cubitus posterior, **L1, L2, L3** sclerites of the left phallomere, **L2v**L2 ventral, **L2vm** median sclerite, **M** media, **Pcu** postcubitus, **R** radius, **RA** radius anterior, **RP** radius posterior, **R1, R2, R3** sclerites of the right phallomere, **ScP** subcostal posterior.

***Coloration*.** Body yellowish brown, face yellowish brown (Fig. 10A, B). Antennae and maxillary palpus brown (Fig. 10D). Pronotum yellowish brown, lateral edges hyaline (Fig. 10C). Tegmina light yellowish brown, lateral edges pale (Fig. 10E). Hind wings infuscate, costal field and appendicular field darker than remaining parts (Fig. 10F). Abdominal sterna, legs, and cerci yellowish brown (Fig. 10B).

***Head and thorax*.** The distance between antennal sockets slightly narrower than interocular space. Fifth maxillary palpus nearly oval, slightly thicker and wider than others (Fig. 10D). Pronotum subelliptic, anterior and posterior margins nearly straight (Fig. 10C). Tegmina with slightly indistinct veins; radius posterior veins of hind wings slightly indistinct, without transverse veins between M and CuA (Fig. 10E, F). Front femur Type B_2_ (Fig. 10B). Pulvilli absent, tarsal claws symmetrical.

***Male genitalia*.** Supra-anal asymmetrical, the left paraproct sheet-like, right paraproct extending backward, hooked, and curled at apex with dense spines (Fig. 10G). Subgenital plate sub-rectangular, the center of anterior and interstylar margins nearly straight. Styli long, length about 1/4 of interstylar space (Fig. 10H). L1 subelliptic, thickened at anterior edge, with a curved and long filamentary structure connected. L2v curved, bifurcated at the apex, with a sharp horn. L2vm sheet-like. L3 extremely robust, with long uncinate part and bent at right angles (Fig. 10I). R1 needle-shaped, the proximal part slightly curved (Fig. 10J). R2 irregular, weakly sclerotized, one of R2 with small protrusions. R3 broad, sheet-like (Fig. 10K).

***Female genitalia*.** Supra-anal plate nearly symmetrical. Paraprocts broad, extending to the posterior margin of supra-anal plate. Intercalary sclerite short, sheet-like. Right first valvifer arm extremely robust, lateral edges folded up, fused with crosspiece (Fig. 16A). First valve robust. Second valve small, basally fused. Third valve broad. The anterior margin of anterior arch slightly sclerotized, with a hook-shaped protrusion, hind edge with two transversely finger-like protrusions. Basivalvula irregular, anterior edge curly. Vestibular sclerite sheet-like (Fig. 16A, B). Laterosternal shelf slightly sclerotized, lateral margin nearly straight (Fig. 16C).

##### Distribution.

China (Yunnan).

#### ﻿ Anaplecta paraomei

Taxon classificationAnimaliaBlattodeaAnaplectidae

Zhu & Che sp. nov.

1C00055D-995D-5D2A-9E28-5AAB4EA0B2EC

http://zoobank.org/D8AD2528-06E2-4980-A090-6CC1089F3256

Figures 11 , 16D–F

##### Type material.

***Holotype***: China • male; Guizhou Prov., Dushan County; 25°45.60'N, 107°33.03'E; 7 June 2019; Lu Qiu, Wen-Bo, Deng, leg.; SWU-B-B-A060104.

***Paratypes***: China • 12 males and 4 females, same data as holotype; SWU-B-B-A060105 and 060120.

##### Diagnosis.

This species is very similar to *A.omei*, but can be distinguished as follows: 1) the paraprocts not extending backward in *A.paraomei* sp. nov., while left paraproct extending backward in *A.omei*; 2) the apex of R1 nearly symmetrical in *A.paraomei*, while asymmetrical in *A.omei*; 3) intercalary sclerite nearly strip-shaped in *A.paraomei*, while spindle-shaped in *A.omei*; and 4) posterior margin of anterior arch hip-shaped in *A.paraomei* sp. nov., while smooth in *A.omei*.

##### Etymology.

The Latin word *para* means similar, referring to its close resemblance to *A.omei*.

##### Measurements (mm).

Male: pronotum length × width: 1.29–1.35 × 2.00–2.09, tegmina length: 5.24–5.53, overall length: 6.15–6.57. Female: pronotum length × width: 1.44 × 2.09, tegmina length: 5.31, overall length: 6.23

##### Description.

**Figure 11.**
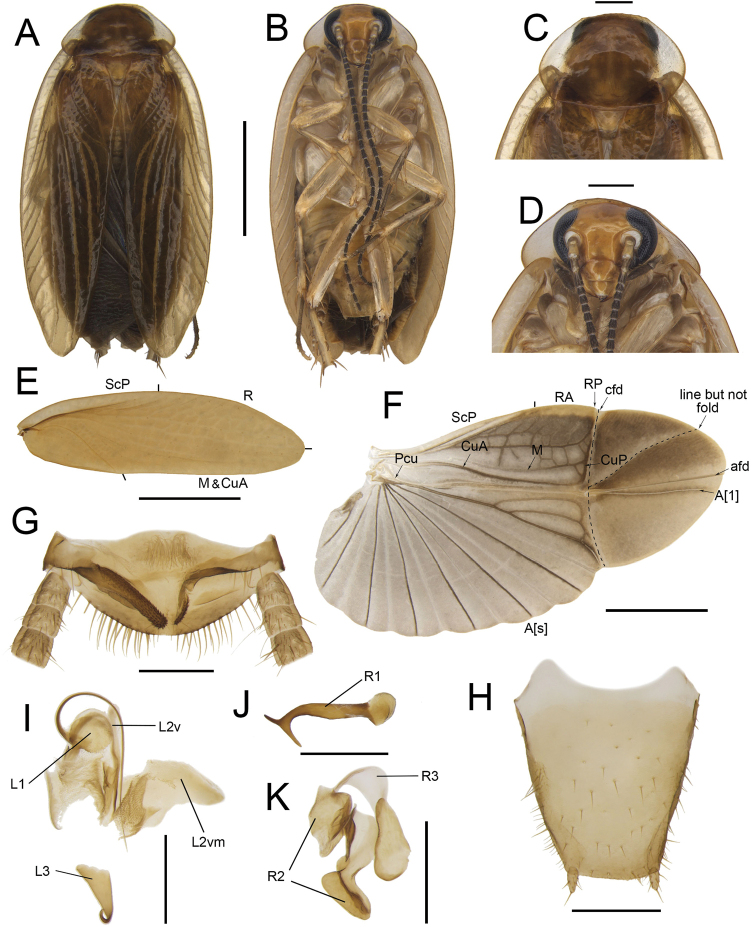
*Anaplectaparaomei* Zhu & Che, sp. nov. holotype (GZ2), male SWU-B-B-A060104 **A** habitus, dorsal view **B** habitus, ventral view **C** pronotum, dorsal view **D** head, ventral view **E** tegmina **F** wings **G** supra-anal plate, ventral view **H** subgenital plate, dorsal view **I** left phallomere, dorsal view **J, K** right phallomere **J** dorsal view **K** ventral view. Scale bars: 2 mm (**A–F**); 0.5 mm (**G–K)**. Abbreviations: **afd** anal fold, **A[1**] the anterior one of the anal vein, **A[s**] the other element of vannal vein, **cfd** cubitus fold, **CuA** cubitus anterior, **CuP** cubitus posterior, **L1, L2, L3** sclerites of the left phallomere, **L2v**L2 ventral, **L2vm** median sclerite, **M** media, **Pcu** postcubitus, **R** radius, **RA** radius anterior, **RP** radius posterior, **R1, R2, R3** sclerites of the right phallomere, **ScP** subcostal posterior.

***Coloration*.** Body yellowish brown, face yellow (Fig. 11A, B). Antennae and maxillary palpus brown (Fig. 11D). Pronotum and tegmina yellowish brown, lateral edges hyaline (Fig. 11C, E). Hind wings infuscate, costal field and appendicular field darker than remaining parts (Fig. 11F). Abdominal sterna, legs, and cerci yellow brown (Fig. 11B).

***Head and thorax*.** The distance between antennal sockets narrower than interocular space. Fifth maxillary palpus nearly oval, slightly thicker and wider than others (Fig. 11D). Pronotum subelliptic, anterior and posterior margins nearly straight, lateral margin protruding and arc-shaped (Fig. 11C). Tegmina with slightly indistinct veins, radius posterior veins of hind wings slightly indistinct, with one transverse vein between M and CuA (Fig. 11E, F). Front femur Type B_2_ (Fig. 11B). Pulvilli absent, tarsal claws symmetrical.

***Male genitalia*.** Supra-anal plate asymmetrical, the left paraproct with dense spines on curly posterior margin; right paraproct with dense spines on curly apex (Fig. 11G). Subgenital plate sub-trapezoidal, the center of anterior slightly curved, interstylar margins straight. Styli medium, length about 1/5 of interstylar space (Fig. 11H). L1 subcircular, with a curved and long filamentary structure. L2v curved, bifurcated at the apex, with a sharp horn. L2vm broad. L3 robust, with extremely bent and sharp uncinate part (Fig. 11I). R1 highly sclerotized, the proximal part nearly dichotomous branching (Fig. 11J). R2 irregular, weakly sclerotized. R3 slightly curved, sheet-like (Fig. 11K).

***Female genitalia*.** Supra-anal plate nearly symmetrical. Paraprocts broad, not extending to the posterior margin of supra-anal plate. Intercalary sclerite short, nearly strip-shaped (Fig. 16D, E). Right first valvifer arm robust, curled (Fig. 16D). First valve robust. Second valve small, basally fused. Third valve broad. The anterior margin of anterior arch slightly curled, with a nearly transparent hook-shaped protrusion and the posterior margin hip-shaped. Basivalvula broad, with dense punctuations, the right lateral deeply concave (Fig. 16D). Vestibular sclerite broad, slightly curled, sheet-like. Laterosternal shelf slightly sclerotized, lateral margin nearly straight. (Fig. 16F).

##### Distribution.

China (Guizhou).

#### ﻿ Anaplecta condensa

Taxon classificationAnimaliaBlattodeaAnaplectidae

Zhu & Che sp. nov.

76AF6B8D-6477-574A-8A44-785CF7C84CA2

http://zoobank.org/92D48955-FA05-41A2-8B3D-E51ABF2A102C

**Figure 12.**
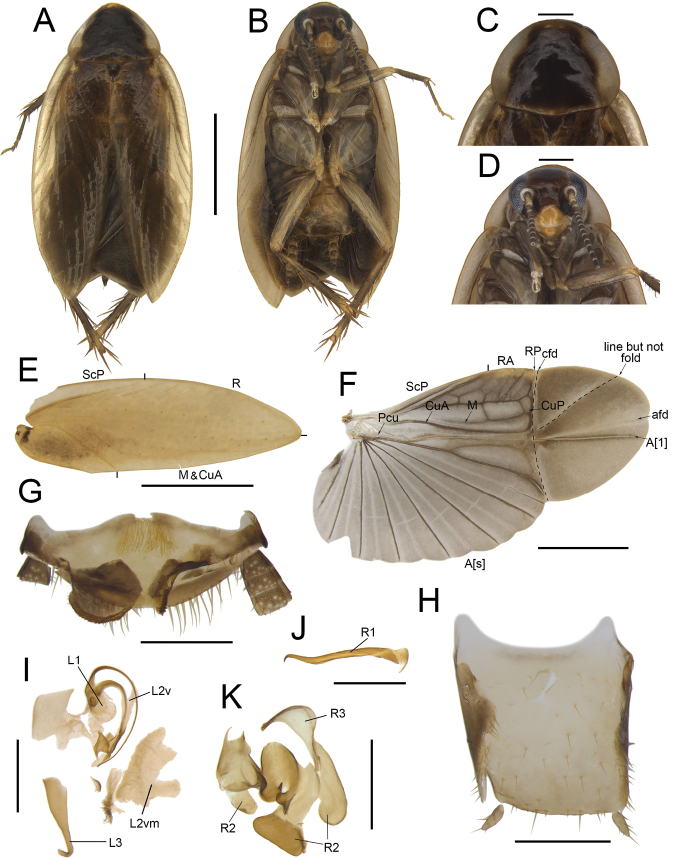
*Anaplectacondensa* Zhu & Che, sp. nov. holotype (GZ4), male SWU-B-B-A060121 **A** habitus, dorsal view **B** habitus, ventral view **C** pronotum, dorsal view **D** head, ventral view **E** tegmina **F** wings **G** supra-anal plate, ventral view **H** subgenital plate, dorsal view **I** left phallomere, dorsal view **J, K** right phallomere **J** dorsal view **K** ventral view. Scale bars: 2 mm (**A–F**); 0.5 mm (**G–K)**. Abbreviations: **afd** anal fold, **A[1**] the anterior one of the anal vein, **A[s**] the other element of vannal vein, **cfd** cubitus fold, **CuA** cubitus anterior, **CuP** cubitus posterior, **L1, L2, L3** sclerites of the left phallomere, **L2v**L2 ventral, **L2vm** median sclerite, **M** media, **Pcu** postcubitus, **R** radius, **RA** radius anterior, **RP** radius posterior, **R1, R2, R3** sclerites of the right phallomere, **ScP** subcostal posterior.

Figures 2C, D , 12 , 16G–I

##### Type material.

***Holotype***: China • male; Guizhou Prov., Libo County, Jiaou Village; 25°30.06'N, 107°67.02'E; 11 June 2019; Lu Qiu, Wen-Bo, Deng, leg.; SWU-B-B-A060121.

***Paratypes***: China • 3 males and 1 female; same data as holotype; SWU-B-B-A060122 to 060125 • 2 males; Guangxi Prov., Guiping City; 31 May–2 June 2014; Shun-Hua Gui, Xin-Ran Li, Jian-Yue Qiu, leg.; SWU-B-B-A060126 and 060127.

##### Diagnosis.

This species is very similar to *A.omei*, but can be distinguished as follows: 1) paraprocts both extending backward in *A.condensa* sp. nov., while only the left extending backward in *A.omei*; 2) R1 needle-shaped in *A.condensa* sp. nov., while bifurcated in *A.omei*; and 3) intercalary sclerite of *A.condensa* sp. nov. very small, filamentous, while that of *A.omei* is spindle-shaped.

##### Etymology.

The specific epithet is derived from the Latin word *condensus*, referring to the paraprocts with dense spines on curly posterior margin.

##### Measurements (mm).

Male: pronotum length × width: 1.36–1.39 × 1.78–1.84, tegmina length: 4.93–5.39, overall length: 5.92–6.59. Female: pronotum length × width: 1.29 × 1.73, tegmina length: 4.75, overall length: 5.82

##### Description.

***Coloration*.** Body brown (some individuals from Guiping yellowish brown) (Fig. 2C, D), face dark brown (Fig. 12A, B). Antennae and maxillary palpus brown (Fig. 12D). Pronotum dark brown, lateral edges nearly hyaline (Fig. 12C). Tegmina yellowish brown, anal field and base of mediocubital field slightly darker (Fig. 12E). Hind wings infuscate, costal field and appendicular field darker than remaining parts (Fig. 12F). Center of abdominal sterna yellow, gradually darkening to dark brown to edges, legs, and cerci dark yellowish brown (Fig. 12B).

***Head and thorax*.** The distance between antennal sockets slightly narrower than interocular space. Fifth maxillary palpus nearly oval, slightly thicker and wider than others (Fig. 12D). Pronotum semicircular, anterior margin arched, the center of posterior margin protrudes slightly (Fig. 12C). Tegmina with indistinct veins, radius posterior veins of hind wings slightly indistinct, without transverse veins between M and CuA (Fig. 12E, F). Front femur Type B_2_ (Fig. 12B). Pulvilli absent, tarsal claws symmetrical.

***Male genitalia*.** Paraprocts both extend backwards and with dense spines on curly posterior margin (Fig. 12G). Subgenital plate sub-rectangular, the center of anterior and interstylar margins nearly straight. Styli long, so is the distance between them (Fig. 12H). L1 subcircular, with curved and long filamentary structure. L2v curved, bifurcated, with a sharp horn. L2vm broad. L3 extremely robust, uncinate part blunt (Fig. 12I). R1 needle-shaped, the proximal part slightly curved (Fig. 12J). R2 irregular, weakly sclerotized. R3 slightly curved, sheet-like (Fig. 12K).

***Female genitalia*.** Supra-anal plate nearly symmetrical, very blunt and round. Paraprocts broad, hind margin blunt, not extending to the posterior margin of supra-anal plate. Intercalary sclerite small, nearly filamentous. First valve robust. Second valve small, basally fused. Third valve broad. The anterior margin of anterior arch slightly curled, with a hook-shaped protrusion (Fig. 16G–H). Basivalvula broad, with dense punctuations, except for left lateral and anterior margin (Fig. 16G). Vestibular sclerite broad, slightly curled, sheet-like. Laterosternal shelf slightly sclerotized, lateral margin straight (Fig. 16I).

##### Distribution.

China (Guizhou, Guangxi).

#### ﻿ Anaplecta cruciata

Taxon classificationAnimaliaBlattodeaAnaplectidae

Deng & Che, 2020

65996EB5-FDB7-525D-AE0A-A18936D906A4

Figure 13J–L

Anaplecta cruciata Deng & Che in Deng et al., 2020: 95–97.

##### Material examined.

China • 8 males (paratypes) and 4 females (paratypes); Yunnan Prov., Xishuangbanna, Mengla County, Yaoqu Town; 21°14.60'N, 101°42.43'E; 820 m; 10 May 2015; Jian –Yue Qiu, leg.; SWU-B-B-A060128 to 060139 • 4 males; Yunnan Prov., Pu’er City, Mt. Wuliang; 24°38'N, 100°44'E; 1232 m; 21 July 2020; Li-Kang Niu, Rong Chen, leg.; SWU-B-B-A060140 to 060143 • 4 males and 3 females; Yunnan Prov., Pu’er City, Meizi Lake; 22°45.27'N, 100°59.60'E; 1365 m; 17 July 2020; Rong Chen, Yi-Shu Wang, leg.; SWU-B-B-A060144 to 060150.

##### Female genitalia.

**Figure 13.**
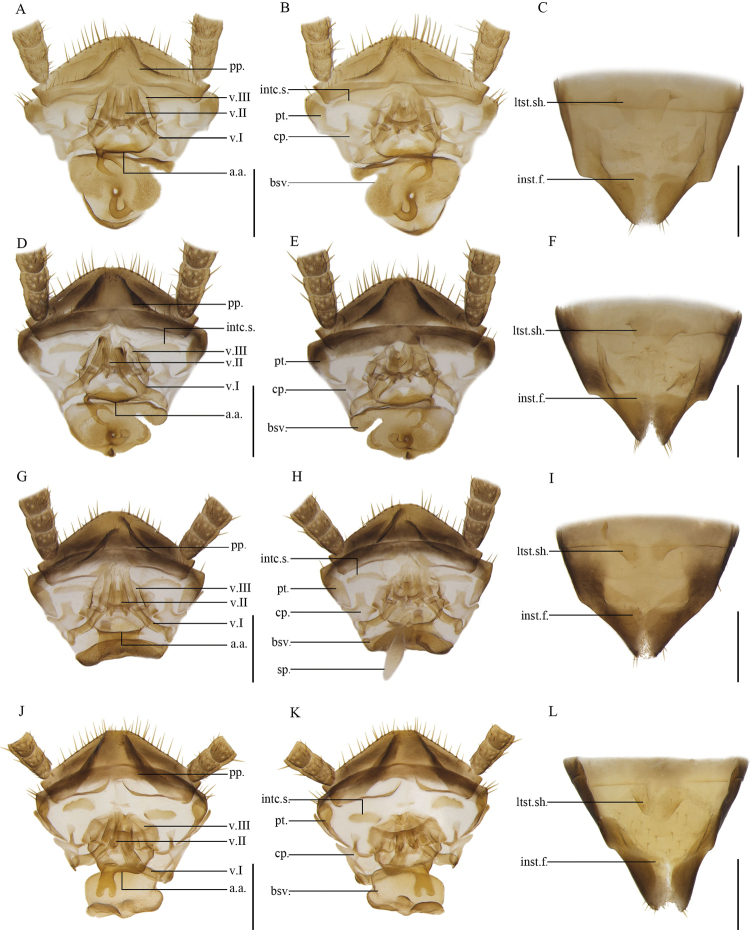
**A–C***Anaplectabicruris* Zhu & Che, sp. nov. paratype, female SWU-B-B-A060004 **D–F***Anaplectaspinosa* Zhu & Che, sp. nov. paratype, female SWU-B-B-A060014 **G–I***Anaplectaserrata* Zhu & Che, sp. nov. paratype, female SWU-B-B-A060020 **J–L***Anaplectacruciata* Deng & Che, 2020. Paratype, female SWU-B-B-A060136 **A, D, G, J** supra-anal plate, ventral view **B, E, H, K** supra-anal plate, dorsal view **C, F, I, L** subgenital plate, dorsal view. Scale bars: 2 mm. Abbreviations: **a.a**. anterior arch, **bsv.** basivalvula, **cp.** crosspiece, **intc.s.** intercalary sclerite, **inst.f.** intersternal fold, **ltst.sh.** laterosternal shelf, **pp.** paraprocts, **pt.** paratergites, **sp.** spermatheca, **v.I** first valve, **v.II** second valve, **v.III** third valve.

Supra-anal plate nearly symmetrical. Paraprocts broad, extending to the posterior margin of supra-anal plate. Intercalary sclerite nearly strip-shaped. First valve robust. Second valve small, basally fused. Third valve broad. The anterior margin of anterior arch slightly sclerotized, protruding forward in a Y-shape. Basivalvula nearly rectangular, with dense punctuations, anterior margin curled (Fig. 13J, K). Laterosternal shelf slightly sclerotized, lateral margin straight (Fig. 13L).

##### Distribution.

China (Yunnan).

#### ﻿ Anaplecta strigata

Taxon classificationAnimaliaBlattodeaAnaplectidae

Deng & Che, 2020

D56E4942-4DFD-523E-9E1E-9B1443AD605E

Figure 14J–L

Anaplecta strigata Deng & Che in Deng et al., 2020: 91–93.

##### Material examined.

China • 11 males and 6 males, Yunnan Prov., Pu’er City, Meizi Lake; 22°45.27'N, 100°59.60'E; 1365 m; 17 July 2020; Rong Chen, Yi-Shu Wang, leg.; SWU-B-B-A060151 to 060167 • 3 females; Yunnan Prov., Xishuangbanna, Shangyong Town; 21°16.19'N, 101°30.42'E; 870 m; 7 July 2020; Du-Ting Jin, Rong Chen leg.; SWU-B-B-A060168 to 060170 • 1 male; Hainan Prov., Linshui County, Mt. Diaoluo; 11 June 2020; Rong Chen, Li-Kang Liu, leg.; SWU-B-B-A060171.

##### Female genitalia.

**Figure 14.**
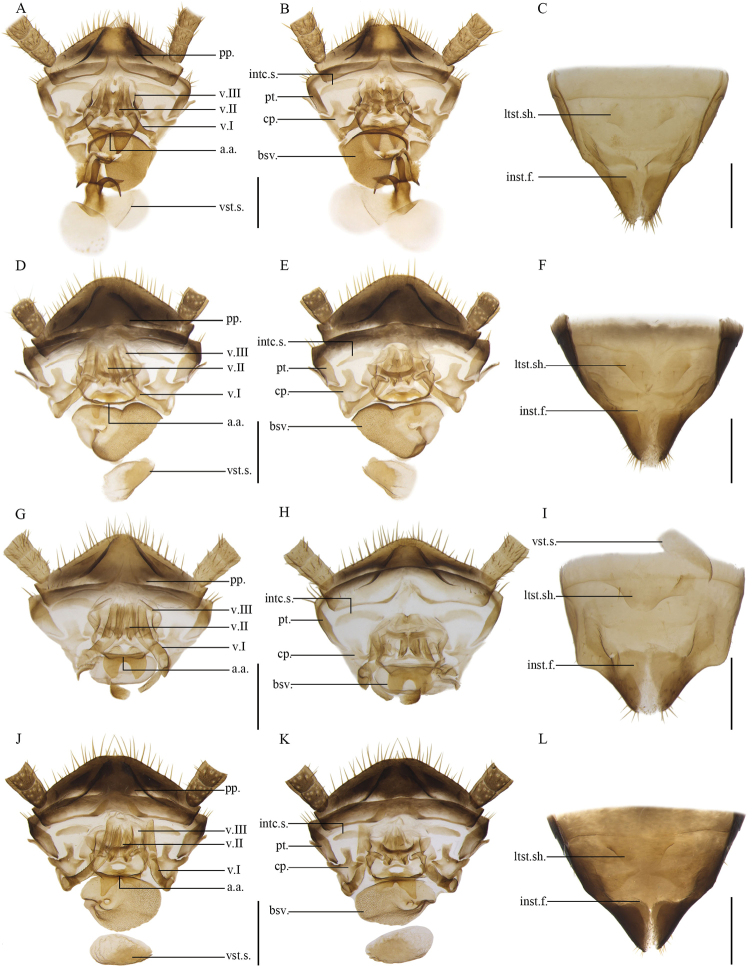
**A–C***Anaplectaungulata* Zhu & Che, sp. nov. paratype, female SWU-B-B-A060034 **D–F***Anaplectaanomala* Zhu & Che, sp. nov. paratype, female SWU-B-B-A060068 **G-I***Anaplectabombycina* Zhu & Che, sp. nov. paratype, female SWU-B-B-A060078 **J-L***Anaplectastrigata* Deng & Che, 2020. Female SWU-B-B-A060168 **A, D, G, J** supra-anal plate, ventral view **B, E, H, K** supra-anal plate, dorsal view **C, F, I, L** subgenital plate, dorsal view. Scale bars: 2 mm. Abbreviations: **a.a**. anterior arch, **bsv.** basivalvula, **cp.** crosspiece, **intc.s.** intercalary sclerite, **inst.f.** intersternal fold, **ltst.sh.** laterosternal shelf, **pp.** paraprocts, **pt.** paratergites, **v.I** first valve, **v.II** second valve, **v.III** third valve, **vst.s.** vestibular sclerite.

Supra-anal plate nearly symmetrical. Paraprocts broad, not extending to the posterior margin of supra-anal plate. Intercalary sclerite strip-shaped. First valve tubular, with scattered erect pubescence. Second valve small, basally fused. Third valve broad. The anterior margin of anterior arch slightly sclerotized, extending forward into two irregular protrusions. Basivalvula approximately triangular, most areas of the basivalvula with dense punctuations. Vestibular sclerite sheet-like, slightly curled (Fig. 14J, K). Laterosternal shelf broad, slightly sclerotized, lateral margin slightly curved (Fig. 14L).

##### Distribution.

China (Hainan, Yunnan).

#### ﻿ Anaplecta basalis

Taxon classificationAnimaliaBlattodeaAnaplectidae

Bey-Bienko, 1969

7F142722-3488-54DC-856D-CB1CA294B747

Figure 15D–F

Anaplecta basalis Bey-Bienko, 1969: 839; Deng et al., 2020: 101.

##### Material examined.

China • 10 males and 7 females; Yunnan Prov., Mengla County, Menglun Town; 21°54.96'N, 101°14.53'E; 624 m; 27 April 2019; Zi-Long Bai, Zhi-Gang Chen leg.; SWU-B-B-A060172 to 060188 • 1 female, Yunnan Prov., Xishuangbanna, Ya’nuo Village; 21°59.70'N, 101°6.02'E; 1212 m; 14 July 2020; Du-Ting Jin, Yi-Shu Wang leg.; SWU-B-B-A060189 • 2 females; Yunnan Prov., Xishuangbanna, Guanping Village; 21°59.06'N, 101°64.40'E; 870 m; 14 July 2020; Rong Chen, Li-Kang Niu leg.; SWU-B-B-A060190 and 060191.

##### Female genitalia.

**Figure 15.**
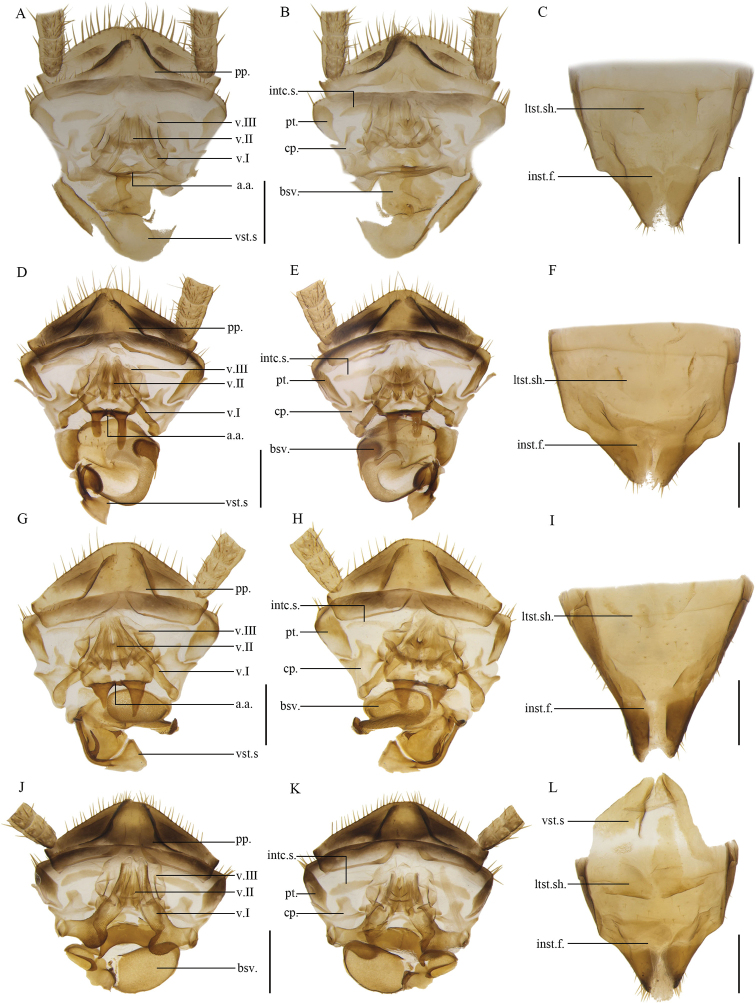
**A–C***Anaplectatruncatula* Zhu & Che, sp. nov. paratype, female SWU-B-B-A060094 **D–F***Anaplectabasalis* Bey-Bienko, 1969. Female SWU-B-B-A060182 **G–I***Anaplectanigra* Deng & Che, 2020. Paratype, female SWU-B-B-A060193 **J–L***Anaplectabicolor* Deng & Che, 2020. Paratype, female SWU-B-B-A060195 **A, D, G, J** supra-anal plate, ventral view **B, E, H, K** supra-anal plate, dorsal view **C, F, I, L** subgenital plate, dorsal view. Scale bars: 2 mm. Abbreviations: **a.a**. anterior arch, **bsv.** basivalvula, **cp.** crosspiece, **intc.s.** intercalary sclerite, **inst.f.** intersternal fold, **ltst.sh.** laterosternal shelf, **pp.** paraprocts, **pt.** paratergites, **v.I** first valve, **v.II** second valve, **v.III** third valve, **vst.s.** vestibular sclerite.

Supra-anal plate nearly symmetrical. Paraprocts broad, extending to the posterior margin of supra-anal plate. Intercalary sclerite slender, long strip-shaped. First valve long. Second valve small, basally fused. Third valve broad. The anterior margin of anterior arch with two highly sclerotized strips (Fig. 15D, E). Basivalvula highly irregular, hind margin slightly curled, with sparse spines, both left and right sides with a brush-like structure (Fig. 15D), the area with punctuations nearly C-shaped (Fig. 15E). Vestibular sclerite irregular, hind margin with two long spines (Fig. 15D). Laterosternal shelf almost hyaline, lateral margin straight (Fig. 15F).

##### Distribution.

China (Yunnan).

#### ﻿ Anaplecta nigra

Taxon classificationAnimaliaBlattodeaAnaplectidae

Deng & Che, 2020

61B38F3B-B592-5AEE-BCB2-5DD89248D7C5

Figure 15G–I

Anaplecta nigra Deng & Che in Deng et al., 2020: 97–99.

##### Material examined.

China • 1 male (holotype) and 1 female (paratype); Xizang Prov., Linzhi City, Motuo County; 29°12.98'N, 95°10.23'E; 1822 m; 16 July 2016; Jian-Yue Qiu, Hao Xu leg.; SWU-B-B-A060192 and 060193.

##### Female genitalia.

Supra-anal plate nearly symmetrical. Paraprocts broad, not extending to the posterior margin of supra-anal plate. Intercalary sclerite slender. First valve long. Second valve small, basally fused. Third valve broad. The anterior margin of anterior arch slightly sclerotized, extending forward to form two elongated triangles protruding. Basivalvula irregular, curled, with dense punctuations. Vestibular sclerite irregular, hind margin with two long spines (Fig. 15G, H). Laterosternal shelf broad, slightly sclerotized, lateral margin straight (Fig. 15I).

##### Distribution.

China (Xizang).

#### ﻿ Anaplecta bicolor

Taxon classificationAnimaliaBlattodeaAnaplectidae

Deng & Che, 2020

4490F323-16CC-5C80-BE95-A158E14C6B05

Figure 15J–L

Anaplecta bicolor Deng & Che in Deng et al., 2020: 99–101.

##### Material examined.

China • 1 male (holotype) and 1 female (paratype); Yunnan Prov., Xishuangbanna, Mengla County; 21°37.33'N, 101°35.28'E; 733 m; 23 May 2016, Lu Qiu, Zhi-Wei Qiu leg.; SWU-B-B-A060194 and 060195.

##### Female genitalia.

Supra-anal plate nearly symmetrical. Paraprocts broad, extending to the posterior margin of supra-anal plate. Intercalary sclerite nearly strip-shaped, tapering to inside. First valve robust, finger-like protrusions on the inner edge with dense spines. Second valve small, basally fused. Third valve broad. The anterior margin of anterior arch protrudes forward in a flaky shape, slightly sclerotized, with an angular protrusion. Basivalvula highly irregular, most areas of the basivalvula with dense punctuations, the rest part curled (Fig. 15J, K). Vestibular sclerite sheet-like. Laterosternal shelf broad, slightly sclerotized, lateral margin straight (Fig. 15L).

##### Distribution.

China (Yunnan).

#### ﻿ Anaplecta omei

Taxon classificationAnimaliaBlattodeaAnaplectidae

Bey-Bienko, 1958

6ED1959C-28A0-5C34-89FC-B4897388AA32

Figure 16J–L

Anaplecta omei Bey-Bienko, 1958: 591; Deng et al., 2020: 101.

##### Material examined.

China • 2 males; Guangxi Prov., Guiping City; 31 May–2 June 2014; Shun-Hua Gui, Xin-Ran Li, Jian-Yue Qiu, leg.; SWU-B-B-A060196 and 060197 • 8 males and 12 females; Guizhou Prov., Tongren City, Mt. Fanjing; 27°70.28'N, 108°84.55'E; 13–14 June 2019; Shu-Ran Liao, Jia-Jun He leg.; SWU-B-B-A060198 to 060217 • 9 males and 3 females; Guizhou Prov., Guiyang City; 26°55.32'N, 106°76.47'E; 6 June 2019, Wen-Bo Deng, Lu-Qiu leg.; SWU-B-B-A060218 to 060229 • 11 males and 22 females; Sichuan Prov., Mt. Omei; 1–5 June 2013; Jin-Jin Wang, Yang Li leg.; SWU-B-B-A060230 to 060262 • 6 males; Guangdong Prov., Zhaoqing City, Mt. Qilin; 23°29.50'N, 109°59.56'E; 8 June 2019; Rong Chen leg.; SWU-B-B-A060263 to 060268 • 3 males and 2 females; Hunan Prov., Mt. Mang; 11–12 July 2015; Zhi-Wei Qiu, Yong-Quan Zhao leg.; SWU-B-B-A060269 to 060273 • 31 males, 9 females; Chongqing City, Youyang County; 29°43.16'N, 109°28.37'E, 30 June 2019, Rong Chen, Hao Xu leg. SWU-B-B-A060274 to 060313 • 40 males, 31 females, Chongqing City, Beibei District; 2018–2019, laboratory staff, leg. SWU-B-B-A060314 to 060384 • 13 males and 1 female; Jiangxi Prov., Lushan City, Mt. Huanglong; 1–2 June 2017, Xin-Ran Li, Li-Li Wang, leg.; SWU-B-B-A060385 to 060398 • 1 male, Zhejiang Prov., Jiangshan City, Shuangxikou Village; 26–27 May 2017; Xin-Ran Li, Li-Li Wang, leg.; SWU-B-B-A060399.

##### Female genitalia.

**Figure 16.**
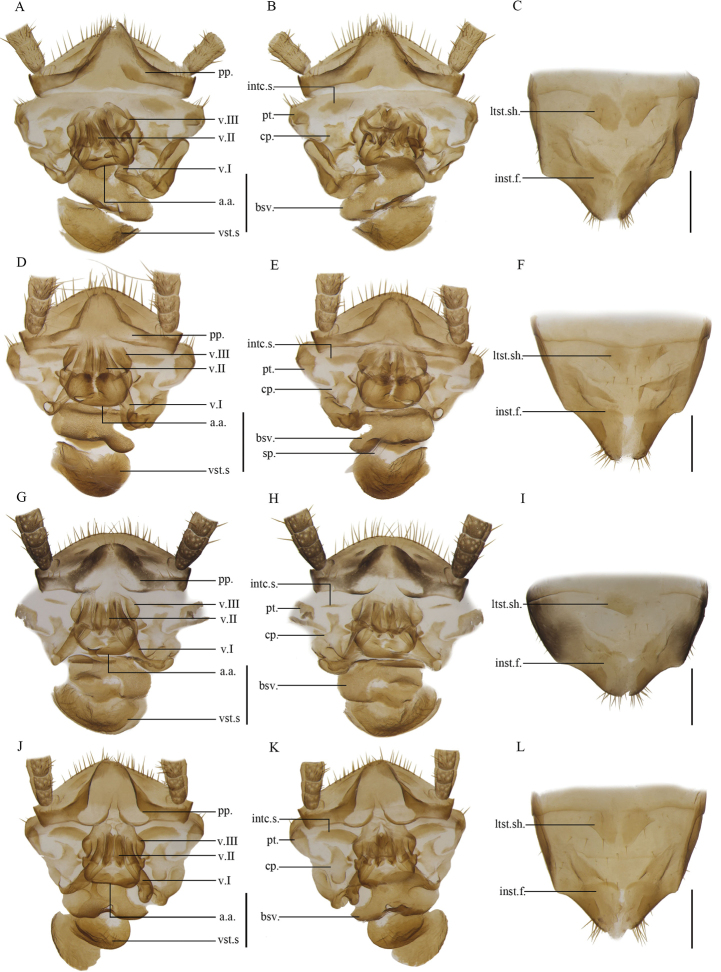
**A–C***Anaplectalongihamata* Zhu & Che, sp. nov. paratype (ZWLS2), female SWU-B-B-A060099 **D–F***Anaplectaparaomei* Zhu & Che, sp. nov. paratype (DS4_2), female SWU-B-B-A060117 **G–I***Anaplectacondensa* Zhu & Che, sp. nov. paratype (GZ10), female SWU-B-B-A060125. **J–L***Anaplectaomei* Bey-Bienko, 1958 (CQ5) female SWU-B-B-A060354 **A, D, G, J** supra-anal plate, ventral view **B, E, H, K** supra-anal plate, dorsal view **C, F, I, L** subgenital plate, dorsal view. Scale bars: 2 mm. Abbreviations: **a.a**. anterior arch, **bsv.** basivalvula, **cp.** crosspiece, **intc.s.** intercalary sclerite, **inst.f.** intersternal fold, **ltst.sh.** laterosternal shelf, **pp.** paraprocts, **pt.** paratergites, **v.I** first valve, **v.II** second valve, **v.III** third valve, **vlf.Ia** first valvifer arm, **vst.s.** vestibular sclerite.

Supra-anal plate nearly symmetrical. Paraprocts broad, extending to the posterior margin of supra-anal plate. Intercalary sclerite short, nearly strip-shaped, slightly curved. One of first valvifer arm robust and curled. First valve robust. Second valve small, basally fused. Third valve broad. The anterior margin of anterior arch slightly curled, with a nearly transparent hook-shaped protrusion. Basivalvula broad, most areas with dense punctuations. Vestibular sclerite broad, slightly curled, sheet-like (Fig. 16J, K). Laterosternal shelf slightly sclerotized, lateral margin slightly curved (Fig. 16L).

##### Distribution.

China (Anhui, Fujian, Jiangsu, Yunnan, Sichuan, Guizhou, Guangdong, Guangxi, Hunan, Chongqing, Zhejiang).

#### ﻿ Anaplecta corneola

Taxon classificationAnimaliaBlattodeaAnaplectidae

Deng & Che, 2020

EB971BD9-0292-53BB-83ED-6A9831221BD5

Figure 17A–C

Anaplecta corneola Deng & Che in Deng et al., 2020: 84–86.

##### Material examined.

China • 20 males and 16 females; Hainan Prov., Ledong County, Mt. Jianfengling, Mingfeng Valley; 18°43.43'N, 108°48.45'E; 579 m; 21–28 May 2014; Shun-Hua Gui, Xin-Ran Li leg.; SWU-B-B-A060400 to 060435 • 14 males and 7 females; Hainan Prov., Ledong County, Mt. Jianfengling; 18°42.63'N, 108°52.75E; 940–960 m; 22–23 June 2020; Yong Li, Jing Zhu leg.; SWU-B-B-A060436 to 060456 • 1 male, Hainan Prov., Qiongzhong County, Mt. Limu; 19°110.59'N, 109°43.77'E; 650 m; 20 June 2020; Yong Li, Jing Zhu, leg.; SWU-B-B-A060457 • 1 female; Hainan Prov., Baisha County, Mt, Yinggeling; 19°04.79'N, 109°123.14'E; 352 m; 18 June 2020; Yong Li, Jing Zhu leg.; SWU-B-B-A060458.

##### Female genitalia.

**Figure 17.**
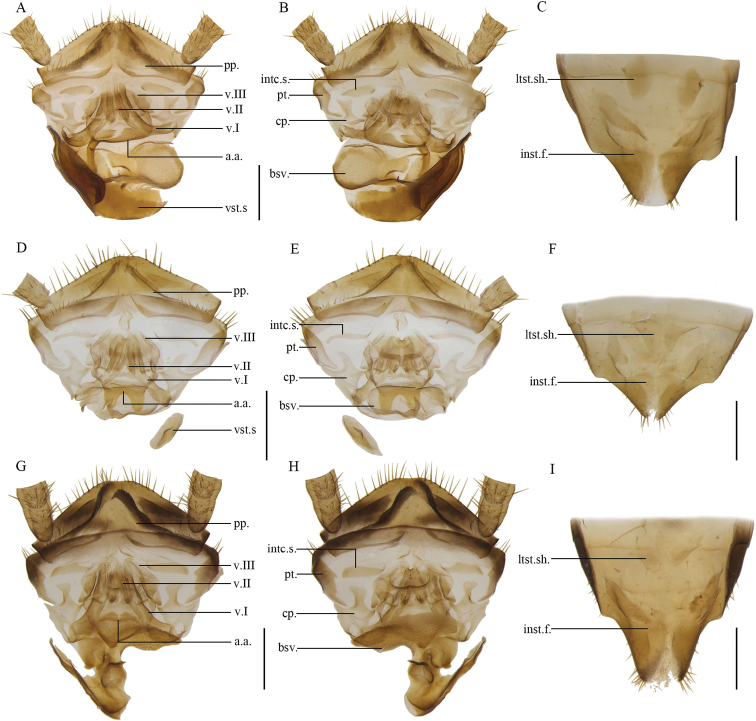
**A–C***Anaplectacorneola* Deng & Che, 2020. Female SWU-B-B-A060450 **D–F***Anaplectaarcuata* Deng & Che, 2020. Female SWU-B-B-A060460 **G–I***Anaplectastaminiformis* Deng & Che, 2020. Paratype, female SWU-B-B-A060462 **A, D, G, J** supra-anal plate, ventral view **B, E, H, K** supra-anal plate, dorsal view **C, F, I, L** subgenital plate, dorsal view. Scale bars: 2 mm. Abbreviations: **a.a**. anterior arch, **bsv.** basivalvula, **cp.** crosspiece, **intc.s.** intercalary sclerite, **inst.f.** intersternal fold, **ltst.sh.** laterosternal shelf, **pp.** paraprocts, **pt.** paratergites, **v.I** first valve, **v.II** second valve, **v.III** third valve, **vst.s.** vestibular sclerite.

Supra-anal plate nearly symmetrical. Paraprocts broad, extending to the posterior margin of supra-anal plate. Intercalary sclerite nearly strip-shaped, tapering to sides. First valvifer arm short. First valve robust. Second valve small, basally fused. Third valve broad. The anterior margin of anterior arch slightly sclerotized, with a near cylindrical protrusion and dense tiny punctuations (Fig. 17A, B). Basivalvula irregular, the right part with dense punctuations, the left anterior margin extending posteriorly to crosspiece (Fig. 17A). Laterosternal shelf slightly sclerotized lateral margin straight (Fig. 17C).

##### Distribution.

China (Fujian, Guangdong, Hainan, Hunan).

#### ﻿ Anaplecta arcuata

Taxon classificationAnimaliaBlattodeaAnaplectidae

Deng & Che, 2020

184BB627-9E4A-594F-9246-E35E672DBA20

Figure 17D–F

Anaplecta arcuata Deng & Che in Deng et al., 2020: 89–90.

##### Material examined.

China • 1 male and 1 female; Hainan Prov.; Qiongzhong County, Mt. Limu; 19°110.59'N, 109°43.77'E; 650 m; 20 June 2020; Rong Chen, Li-Kang Niu, leg.; SWU-B-B-A060459 and 060460.

##### Male genitalia.

On the basis of careful observation, we suspect that the L2d mentioned by Deng et al. (2020) may be the degraded right phallomere.

##### Female genitalia.

Supra-anal plate nearly symmetrical. Paraprocts broad, not extending to the posterior margin of supra-anal plate. Intercalary sclerite slender. First valve robust. Second valve small, basally fused. Third valve broad. The anterior margin of anterior arch slightly sclerotized, extending forward in a flaky shape with a deep concave in the middle. Basivalvula nearly elliptic with dense punctuations. Vestibular sclerite sheet-like (Fig. 17D, E). Laterosternal shelf slightly sclerotized, lateral margin slightly curved (Fig. 17F).

##### Distribution.

China (Hainan).

#### ﻿ Anaplecta staminiformis

Taxon classificationAnimaliaBlattodeaAnaplectidae

Deng & Che, 2020

B6816C8A-A450-589F-92F2-D5B497498D94

Figure 17G–I

Anaplecta staminiformis Deng & Che in Deng et al., 2020: 86–88.

##### Material examined.

China • 1 male (holotype) and 1 female (paratype); Hainan Prov., Linshui County, Mt. Diaoluo; 18°28.50'N, 109°31.87'E; 423 m; 16 April 2015; Lu Qiu, Qi-Kun Bai leg.; SWU-B-B-A060461 and 060462 • 2 males (paratypes) and 4 females (paratypes); Hainan Prov., Ledong County, Mt. Jianfengling, Mingfeng Valley; 18°25.95'N, 108°28.96'E; 579 m; 18 May 2014; Shun-Hua Gui, Xin-Ran Li leg.; SWU-B-B-A060463 to 060468.

##### Female genitalia.

Supra-anal plate nearly symmetrical. Paraprocts broad, not extending to the posterior margin of supra-anal plate. Intercalary sclerite nearly strip-shaped, tapering to insides. First valve robust. Second valve small, basally fused. Third valve broad. The anterior margin of anterior arch slightly sclerotized, extending forward in a heart shape, with a nodular protrusion at apex (Fig. 17G, H). Basivalvula irregular, the left anterior margin extending posteriorly to first valvifer arm, deep depression in the center, posterior margin broad with dense punctuations (Fig. 17G). Laterosternal shelf slightly sclerotized, lateral margin slightly curved (Fig. 17I).

##### Distribution.

China (Hainan).

#### ﻿ Anaplecta furcata

Taxon classificationAnimaliaBlattodeaAnaplectidae

Deng & Che, 2020

5F357034-790E-5AC1-9C65-BCD07A17348F

Anaplecta furcata Deng & Che in Deng et al., 2020: 93–95.

##### Material examined.

China • 2 males (paratypes); Guangxi Prov., Jinxiu County, Mt Dayao; 24°8.43'N, 110°11.70'E; 944 m; 7 July 2015; Lu Qiu, Qi-Kun Bai leg.; SWU-B-B-A060469 and 060470

##### Distribution.

China (Guangxi).

## ﻿Discussion

In recent years, male genitalia were the main characteristics in the species delimitation of *Anaplecta* (Lucañas, 2016; Deng et al. 2020) but DNA barcodes can also help to delimit and distinguish species (Deng et al. 2020). During examination of samples of *Anaplectaomei*, we found some subtle morphological differences between samples collected in Libo, Dushan, Mt. Wuliang, and other regions. This included color, paraprocts, subgenital plates, and phallomeres. Although these specimens were recovered as four MOTUs in ABGD, these subtle differences in morphology were insufficient to determine whether they reflect intraspecific variation or interspecific differences. Therefore, we turned to the female genitalia for more evidence. Surprisingly, we found the shapes of first valvifer arm, intercalary sclerite, anterior arch, and basivalvula were stable within these four MOTUs and differed between MOTUs. Khalifa (1950) mentioned that when a pair of *Blattellagermanica* mated, the hooked left phallomere (L3) extended and secured the first valve allowing the male to physically attach to the female during copulation. Therefore, we hypothesize that the long and robust hook of male genitalia of SP4 is to match the robust first valvifer arm of its female. Graves (1969) speculated that when transferring the spermatophore, the soft outer layer of the spermatophore hardens and would be against the female genital sclerites in order to ensure the openings of the sperm sacs aligned directly with the female spermathecal opening. Thus, we infer that the anterior arch and basivalvula might be related to this process of transferring the spermatophore. Taking all this evidence together, we can consider these MOTUs as different species: *A.longihamata* sp. nov., *A.paraomei* sp. nov., and *A.condensa* sp. nov. Similarly, we also found significant differences in other species in the anterior arch and basivalvula, indicating that the variation in female genitalia can be applied to identify the species of *Anaplecta*. However, this has often been neglected in the past study of *Anaplecta*, with the exception of McKittrick (1964), who described the female genitalia in detail. Only the valvular subgenital plate was involved in other studies (Roth, 1990; Deng et al. 2020). In our study, the characteristics of the female genitalia played an important role in detecting these three cryptic species; hence, researchers should pay more attention to female genitalia in future studies.

## Supplementary Material

XML Treatment for Anaplecta bicruris

XML Treatment for Anaplecta spinosa

XML Treatment for Anaplecta serrata

XML Treatment for Anaplecta ungulata

XML Treatment for Anaplecta anomala

XML Treatment for Anaplecta bombycina

XML Treatment for Anaplecta truncatula

XML Treatment for Anaplecta longihamata

XML Treatment for Anaplecta paraomei

XML Treatment for Anaplecta condensa

XML Treatment for Anaplecta cruciata

XML Treatment for Anaplecta strigata

XML Treatment for Anaplecta basalis

XML Treatment for Anaplecta nigra

XML Treatment for Anaplecta bicolor

XML Treatment for Anaplecta omei

XML Treatment for Anaplecta corneola

XML Treatment for Anaplecta arcuata

XML Treatment for Anaplecta staminiformis

XML Treatment for Anaplecta furcata

## Supplementary materials

Supplementary material 1 Table S1. Pairwise genetic divergence of distances Data type genetic distances Explanation note Pairwise genetic divergence of distances calculated by K2P model method using cytochrome oxidase subunit I (COI) gene sequences in MEGA. https://binary.pensoft.net/file/630060This dataset is made available under the Open Database License (http://opendatacommons.org/licenses/odbl/1.0/). The Open Database License (ODbL) is a license agreement intended to allow users to freely share, modify, and use this Dataset while maintaining this same freedom for others, provided that the original source and author(s) are credited.Jing Zhu
